# A small number of abnormal brain connections predicts adult autism spectrum disorder

**DOI:** 10.1038/ncomms11254

**Published:** 2016-04-14

**Authors:** Noriaki Yahata, Jun Morimoto, Ryuichiro Hashimoto, Giuseppe Lisi, Kazuhisa Shibata, Yuki Kawakubo, Hitoshi Kuwabara, Miho Kuroda, Takashi Yamada, Fukuda Megumi, Hiroshi Imamizu, José E. Náñez Sr, Hidehiko Takahashi, Yasumasa Okamoto, Kiyoto Kasai, Nobumasa Kato, Yuka Sasaki, Takeo Watanabe, Mitsuo Kawato

**Affiliations:** 1 Department of Youth Mental Health, Graduate School of Medicine, The University of Tokyo, Tokyo 113-0033, Japan; 2 Diagnostic Imaging Program, Molecular Imaging Center, National Institute of Radiological Sciences, Chiba 263-8555, Japan; 3 Department of Decoded Neurofeedback, ATR Brain Information Communication Research Laboratory Group, Kyoto 619-0288, Japan; 4 Department of Brain Robot Interface, ATR Brain Information Communication Research Laboratory Group, Kyoto 619-0288, Japan; 5 Medical Institute of Developmental Disabilities Research, Showa University Karasuyama Hospital, Tokyo 157-8577, Japan; 6 Department of Language Sciences, Tokyo Metropolitan University, Tokyo 192-0397, Japan; 7 Department of Cognitive, Linguistic and Psychological Sciences, Brown University, Rhode Island 02912, USA; 8 Department of Child Neuropsychiatry, Graduate School of Medicine, The University of Tokyo, Tokyo 113-0033, Japan; 9 Disability Services Office, The University of Tokyo, Tokyo 113-0033, Japan; 10 Child Mental Health-Care Center, Fukushima University, Fukushima 960-1296, Japan; 11 Institute of Cognitive Neuroscience, University College London, London WC1N 3AZ, UK; 12 Department of Psychology, Graduate School of Humanities and Sociology, The University of Tokyo, Tokyo 113-0033, Japan; 13 School of Social and Behavioral Sciences, Arizona State University, Phoenix, Arizona 85306, USA; 14 Department of Psychiatry, Kyoto University Graduate School of Medicine, Kyoto 606-8507, Japan; 15 Department of Psychiatry and Neurosciences, Graduate School of Biomedical Sciences, Hiroshima University, Hiroshima 734-8553, Japan; 16 Department of Neuropsychiatry, Graduate School of Medicine, The University of Tokyo, Tokyo 113-0033, Japan

## Abstract

Although autism spectrum disorder (ASD) is a serious lifelong condition, its underlying neural mechanism remains unclear. Recently, neuroimaging-based classifiers for ASD and typically developed (TD) individuals were developed to identify the abnormality of functional connections (FCs). Due to over-fitting and interferential effects of varying measurement conditions and demographic distributions, no classifiers have been strictly validated for independent cohorts. Here we overcome these difficulties by developing a novel machine-learning algorithm that identifies a small number of FCs that separates ASD versus TD. The classifier achieves high accuracy for a Japanese discovery cohort and demonstrates a remarkable degree of generalization for two independent validation cohorts in the USA and Japan. The developed ASD classifier does not distinguish individuals with major depressive disorder and attention-deficit hyperactivity disorder from their controls but moderately distinguishes patients with schizophrenia from their controls. The results leave open the viable possibility of exploring neuroimaging-based dimensions quantifying the multiple-disorder spectrum.

## Abstract (web summary)

Autism spectrum disorder (ASD) is manifested by subtle but significant changes in the brain. Here, Yahata and colleagues devise a novel machine learning algorithm and develop a reliable ASD classifier based on brain functional connectivity, with which they quantitatively measure neuroimaging dimensions between ASD and other mental disorders.

## Graphical abstract

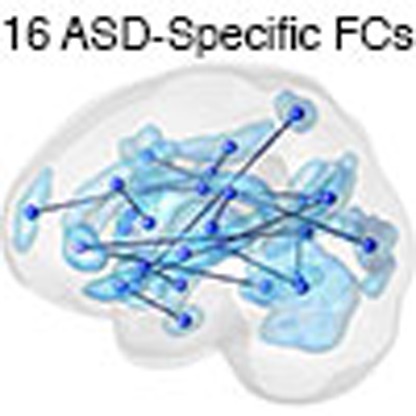

Autism spectrum disorder (ASD) is a major developmental disorder characterized by repetitive, restricted behaviour as well as deficits in communication and reciprocal social interactions^1^. ASD has attracted a great deal of attention of basic and clinical scientists in the hope that clarification of its underlying mechanisms will lead to the development of remedies for ASD as well as a better understanding of the neural substrates of important cognitive functions, including social behaviour^2^. Despite the significance of the disorder, no effective biomarker has been developed. The medical diagnosis for ASD has been made largely based on narrative interactions between individuals and clinical professionals. With the exception of ‘clear and typical' cases, such diagnostic methods without any biological grounds could run the risk of producing a high variance in diagnosis^3^ and delaying the detection of abnormalities^4^.

Magnetic resonance imaging (MRI)-based characterization of ASD has been explored as a complement to the current behaviour-based diagnoses. While previous studies have identified a multitude of ASD-specific structural and functional abnormalities, none of them were actually implemented as a reliable biomarker. The most crucial reason for this disappointing situation^5^,^6^ may be the lack of its generalizability—the validity of the previously developed classifiers has not been established in terms of the diversity of population demographics and the variety of data attributes^7^,^8^,^9^,^10^,^11^,^12^,^13^. These demographics and data attributes include different ethnicities, ages^14^, sex^15^, medication profiles^16^, scanner specifications^17^, imaging parameters^18^ and instructions to participants^19^. All of these aspects are known to affect the MRI data. Without proof of generalization, the classifier can neither be regarded as practical in clinical applications, nor can neuroimaging features selected by the classifier be regarded as the candidate neural substrates of ASD.

The issue surrounding the generalization of neuroimaging-based biomarkers for psychiatric disorders has attracted little attention in neuropsychiatry until recently^20^,^21^. The majority of the previous developments of ASD biomarkers were made based on a single-site data, leaving the generalizability issue out of reach^7^,^8^,^9^,^10^,^11^,^12^,^13^. This situation has held true in more recent investigations that incorporated multiple-site data^22^,^23^,^24^,^25^, for which the generalizability issue was not examined for an independent validation cohort. There is one unsuccessful attempt in which the generalizability of a classifier was tested on an independent validation cohort^26^. This study applied a classifier, that was previously developed based on measures of structural MRI for the population of the UK^7^,^8^, to Japanese ASD individuals^26^. The classifier exhibited more than 80% sensitivity and specificity for the UK training data. However, its performance was no better than chance level for the Japanese test cohort. The results indicate that the development of a reliable neuroimaging-based biomarker is extremely challenging.

To develop a generalizable classifier, we must overcome the following two major difficulties: over-fitting and nuisance variables (NVs). First, particular conditions in data and model properties can cause the over-fitting problem^20^ in which model fitting to the training data can be so accurate that the associated errors become artificially smaller compared with the inherent data variance. This inflated prediction performance typically fails when the model is applied to independent data that are not used for determination of the model. Among other possibilities, determining a large number of model parameters using a relatively small data sample almost inevitably leads to the state of over-fitting, which makes the generalization capability of the model extremely poor. For example, the identification of ASD-specific features in magnetic resonance images must necessarily entail a search over a few 10^4^ to 10^5^ voxels (or its squared number for voxel-to-voxel functional connections (FCs)) using a data set that typically consists of not more than ∼10^3^ individuals. In this case, the derived classification scheme falls almost inevitably to a state of over-fitting, resulting in catastrophic generalization to the external data^20^. In essence, an excessive number of free parameters in the model introduces undesirable fitting to a unique structure of the data, including inherent noise.

Second, any machine-learning algorithm used for classification is doomed to exploit NVs unique to a given sample data, and to erroneously select neuroimaging features that are correlated with the NVs. NVs include both site-specific conditions in image acquisition and properties in the sample population such as demographic attributes, medication status, and onset and duration of illness. However, the neuroimaging features correlated with these NVs are irrelevant to ASD itself in an independent validation cohort, and naturally for the general ASD population. To avoid biased extraction of ASD irrelevant features, it is thus essential that classifier development proceed with a large population that is recruited at multiple sites and that all the possible NVs be identified, controlled and removed appropriately in the feature selection (FS) process.

Here, for the first time we have developed an ASD classifier using a multiple-site data set in Japan, and confirmed its generalization capability in two independent validation cohorts in the USA and Japan. We focus on abnormal functional connectivity (FC) in ASD as revealed by resting-state fcMRI (rs-fcMRI)^6^,^27^,^28^,^29^. To suppress the over-fitting and the effects of NVs, a unique combination of machine-learning algorithms automatically and objectively identifies a small number of FCs related to the ASD-specific abnormality. The resulting ASD classifier, based only on the identified FCs, achieves generalization capability across multiple imaging sites (site generalization).

We also examine a different type of generalizability of the classifier towards other disorders (disorder generalization). It is generally believed that ASD shares aetiological and pathophysiological backgrounds with schizophrenia (SCZ) to a greater degree than with attention-deficit hyperactivity disorder (ADHD) and major depressive disorder (MDD)^30^,^31^,^32^,^33^. Recent genome-level studies reported that ASD shares a significant degree of polygenic risk with SCZ, but not with ADHD or MDD^30^,^31^. An accumulating body of evidence by clinical, behavioural and neuronal phenotypes studies has shown a close relationship between ASD and SCZ^32^,^33^. To our knowledge, no study has quantified spectral relationships among multiple psychiatric disorders with respect to biological dimensions defined by intrinsic FCs. Here we examine the spectral relationships among four disorders, ASD, SCZ, ADHD and MDD, by means of a measure provided by the ASD classifier. More concretely, we examine the extent to which the ASD classifier is specific to ASD or extendable (generalizable) to other psychiatric disorders. This question can be rephrased as follows. Does the ASD classifier discriminate only ASD individuals from TD control (that is, specific to ASD), or does it discriminate patients with general psychiatric disorder from their healthy controls (that is, generalized to other disorders)? At the individual level, the output of the ASD classifier might provide a quantitative measure of ‘ASD-ness' along one of biological dimensions in psychiatric disorders (Supplementary Fig. 1). If the ASD classifier shows specificity and/or generalizability only for a certain range of disorders, the ASD-ness may provide a useful biological dimension across the multiple-disorder spectrum^34^,^35^.

## Results

### Highly accurate Japanese-population-based classifier for ASD

We constructed a classifier based on the FCs of each individual to distinguish ASD from TD adults. The whole brain images were collected from three different sites in Japan (74 high-functioning adult ASDs and 107 adult TDs; see Methods for details). Age and sex were matched between ASD and TD for sites A and B. Site C collected only TD data (Supplementary Table 1). This unbalanced ASD/TD composition among the three sites was taken care of by a machine-learning algorithm as explained below. Each individual image was divided into 140 regions using a sulci-based anatomical atlas (extended Brainvisa Sulci Atlas)^36^. To obtain interregional FCs through rs-fcMRI, Pearson correlation coefficients were computed for all possible 9,730 pairs of these 140 regions from their mean fMRI time-series data. A machine-learning technique was then applied to the whole set of correlation matrices to optimally select a subset of FCs so that the best classification performance would be obtained. Specifically, we applied the *L*_1_-norm regularized sparse canonical correlation analysis (*L*_1_-SCCA)^37^ to the data set to identify a subset of FCs relevant only to the neural substrates of ASD while factoring out the effects of noise and NVs associated with the data. In particular, the unbalanced ASD/TD composition was addressed appropriately by incorporating the site label as NV (see Methods subsection ‘Selecting FCs as the ASD classifier'). We then employed the sparse logistic regression (SLR)^38^ to further perform dimension reduction to mitigate the over-fitting and thereby to extract the essential FCs representing the core abnormal connectivity in ASD (see Methods and Supplementary Fig. 2).

**Figure 1:**
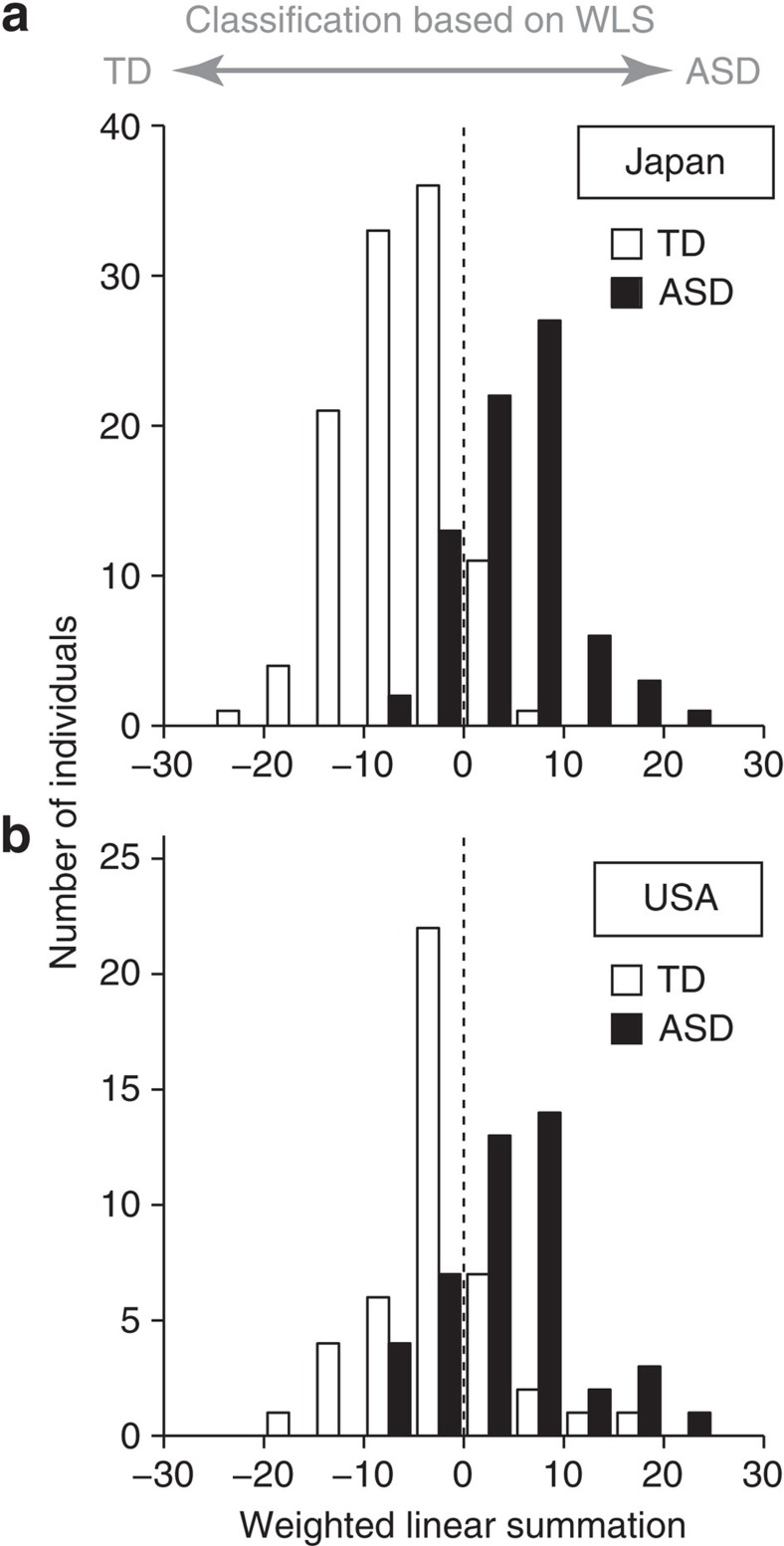
Distribution of weighted linear summations (WLS) of functional connections used for the classification of ASD and TD. (**a**) The number of TD (white) and ASD (black) individuals in the Japanese data included in a specific WLS interval of width 5 is shown as a histogram (see also Supplementary Fig. 5). (**b**) WLS for the US ABIDE dataset in the same formats as **a**.

The classification accuracy was evaluated by the leave-one-participant-out cross validation procedure (LOOCV, see Methods). At each iteration, the classifier incorporated only 15.3±0.7 out of 9,730 FCs (0.2% of the entire FCs). Supplementary Note 1 and Supplementary Fig. 3 show the robustness and stability of the identified FCs across the cross-validation procedure. The classifier separated ASD- from TD-populations with an accuracy of 85% (Permutation test, *P*=0.001; see Supplementary Fig. 4)^39^. The corresponding area under the curve (AUC) was 0.93, indicating high discriminatory ability. For this classifier, the weighted linear summation (WLS or linear discriminant function) of the correlation values of the identified FCs predicted the diagnostic label of each individual. An individual with a positive and negative WLS was classified as ASD and TD, respectively. Figure 1a shows that the two WLS distributions of the ASD and TD populations from the Japanese data set were clearly separated by the threshold of WLS=0, to the right (ASD) and to the left (TD). The sensitivity was 80% and specificity was 89%. This leads to a high diagnostic odds ratio (DOR) of 31.1, which indicates that the effect size is very large. We found that high classification accuracy was not only achieved for the entire data set, but also distributed equally among the three imaging sites in Japan (85% accuracy for all the sites A–C; see Supplementary Fig. 5 and Supplementary Table 2).

### Generalization of the classifier for independent cohorts

We tested the generalizability of the classifier using two independent validation cohorts obtained from the US ABIDE Project^40^ and from site B in Japan (see Methods). In the ABIDE data pool, we selected a subset of individuals that contained 44 high-functioning adults with ASD and demographically matched 44 TD adults, who were recruited from seven sites in this data pool (see Methods). For this US-independent validation cohort located across the Pacific from Japan, the present classifier, trained only with Japanese labelled samples, achieved a high performance with an accuracy of 75% (AUC=0.76) and a DOR of 9.0. The probability of obtaining this high performance by chance is extremely small as *P*=1.4 × 10^−6^ (one-sided binomial test) and *P*=0.001 (permutation test, see Supplementary Fig. 4). We emphasize that this performance was achieved when the classifier was applied to the US ABIDE data set for the first time without any retuning of the machine-learning procedure. Thus, this USA data set was a true and final validation set. One of the reasons for the true validation test is that our algorithm does not allow any parameter tuning based on the validation cohort (see also Methods). As can be seen in the WLS distribution for the USA data shown in Fig. 1b, the degree of separation between the ASD and TD populations was almost comparable to that of the Japanese data set shown in Fig. 1a. This indicates that the present classifier was successfully generalized across the Pacific to an independent validation cohort of more diverse races/ethnicities that were acquired under various imaging settings and conditions. These results indicate that although we developed a highly reliable classifier by only using the training data obtained from Japan, it is sufficiently universal to classify ASD/TD in the USA validation cohort.

Given the possibility that the identification of ASD-specific FCs in the classifier could have been influenced by ASD individuals' factors irrelevant to the core ASD pathology, including secondary symptoms and the medication status, the next challenge was to examine how the ASD classifier is resistant to such complications. By relaxing the selection criteria for ASD, we identified 19 additional individuals with ASD and demographically matched 19 additional TDs in the ABIDE data pool (see Methods and Supplementary Note 2). We appended these individuals into the main USA data set to form the extended ABIDE data set that consisted of 63 individuals with ASDs and 63 TDs. Repeating the same analysis for this broadened data set, we found the results to be: AUC=0.74, accuracy=71% and DOR=6.4, which were slightly worse than the original narrower data set.

We further confirmed the generalizability of the classifier using the Japanese-independent validation cohort (AUC=0.77, accuracy=70%). This cohort incorporated 27 individuals with ASD and 27 demographically matched TDs. These participants were scanned more recently using a newer and different 3 T MR scanner at site B in Japan, compared to the original Japanese discovery cohort.

### Characteristics of the 16 identified FCs in the classifier

**Figure 2:**
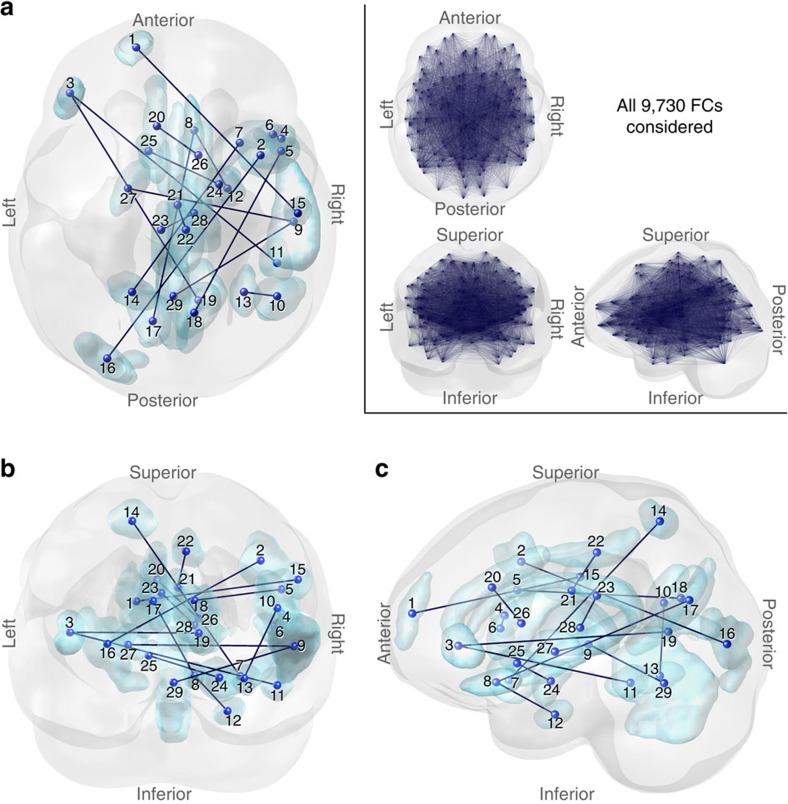
The 16 FCs identified for the ASD/TD classifier. (**a**–**c**) The 16 FCs viewed from (**a**) top, (**b**) posterior and (**c**) left. The inset displays all 9,730 FCs. The 29 terminal regions connected by the 16 FCs were numbered as follows: in the frontal lobe, the superior (1), middle (2), inferior (3, left; 4–7, right) gyri and rectus (8); in the temporal lobe, the superior (9), middle (10), inferior (11), parahippocampal (12) and fusiform (13) gyri; in the parietal lobe, the superior parietal lobule (14) and the postcentral gyrus (15); in the occipital lobe, the middle occipital gyrus (16), cuneus (17, left; 18, right) and the calcarine fissure (19); in the limbic system, the anterior (20), middle (21–22), posterior (23) cingulate gyri and amygdala (24); in the basal ganglia, the caudate (25, left; 26, right), pallidum (27), thalamus (28); and cerebellum (29). See also Table 1 and Supplementary Movie 1.

Figure 2 shows the spatial distribution of the 16 FCs that were automatically and objectively identified from the data for reliable classification of ASD and TD by the machine-learning algorithm. A detailed list of FC properties is provided in Table 1. Because the reliability of classification was generalized to the two independent cohorts, these FCs are thought to be much more trustworthy in characterizing neural substrates of ASD than the FCs that were simply selected in many previous studies by conventional statistical thresholding of ASD/TD differences within a limited data set^41^.

**Figure 3:**
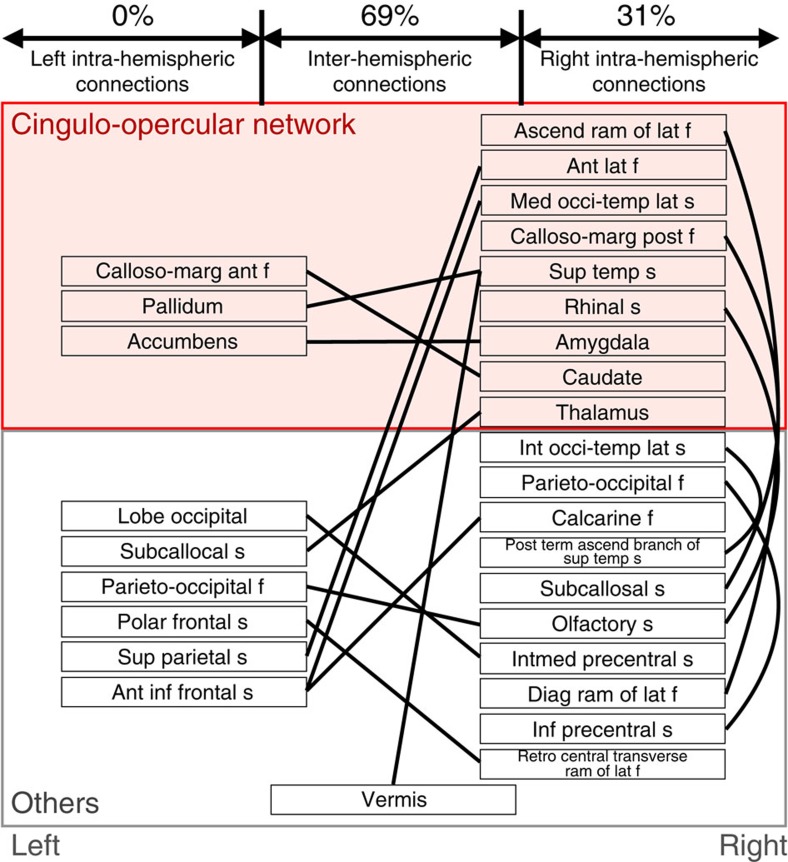
The 16 FCs (solid lines) and their terminal regions (names in boxes). The left and right halves of the figure correspond to the left and right brain hemispheres, respectively. The FCs were classified into three hemispherical categories: left intra-hemispheric, right intra-hemispheric and inter-hemispheric. The terminal regions defined by the Brainvisa Sulci Atlas belong to either cingulo-opercular or other networks. The red background indicates the cingulo-opercular network. ant, anterior; ascend, ascending; calloso-marg, calloso-marginal; diag, diagonal; f, fissure; inf, inferior; int, internal; intmed, intermediate; lat, lateral; med, median; occi-temp, occipito-temporal; post, posterior; ram, ramus; s, sulcus; sup, superior; temp, temporal; term, terminal.

We identified the following three major characteristics of the 16 FCs in terms of their hemispheric distributions and attributions to known intrinsic functional networks (Fig. 3 and Table 1). First, regarding the hemispheric distribution of the FCs, inter-hemispheric (69%) and right intra-hemispheric (31%) FCs dominated, whereas the left intra-hemispheric FCs were absent (one-sided binomial test, *P*=0.01). Second, regarding the hemispheric distribution of the brain regions involved in the 16 FCs, there were significantly more regions in the right hemisphere than in the left (one-sided binomial test, *P*=0.05). Third, regarding the functional network attributes of the 32 brain regions comprising these 16 FCs (allowing for duplicates in the count), 41% (13 regions) belonged to the cingulo-opercular network^14^,^42^. This percentage was significantly higher than 24%, the anatomically expected percentage, given 33 cingulo-opercular regions among a total of 140 regions (=33/140 × 100) (one-sided binomial test, *P*=0.02; see also Table 1).

The state of FC exhibiting the smaller (that is, more negative) and greater (more positive) mean correlation index in the ASD population than the TD control is termed under- and over-connectivity, respectively. In the 16 FCs incorporated in the classifier, ASD exhibited under-connectivity in nine FCs (*r*_ASD_<*r*_TD_) and over-connectivity in seven FCs (*r*_ASD_>*r*_TD_) compared with TD. See Table 1 for the mean correlation values for the ASD and TD populations and Supplementary Fig. 6 for their distributions. A *χ*^2^-test indicated that there was no significant difference between the number of FCs exhibiting under- and over-connectivity (*P*=0.62). These results suggest that neither the under- or over-connectivity hypothesis^43^,^44^,^45^ alone can successfully describe the overall aberrancy in the FCs exhibited by the ASD population. One previous study observed a similar trend for the FCs selected simply by using a statistical threshold for the difference in FC between the two populations^41^. However, there is no guarantee that the FCs that survived should represent the neural substrates of ASD. This is because any statistical result using thresholds varies with a specific data set and the thresholds. Our result is the first demonstration that neither the under- or over-connectivity hypothesis alone sufficiently characterizes the FCs that can classify the ASD population from the TD population in independent validation cohorts (Supplementary Fig. 6 and Table 1). Furthermore, the present findings are at odds with the previous distance-dependent abnormality hypothesis that proposed disrupted long-range and enhanced short-range connectivity in ASD^46^. Specifically, we found that the mean distance of the FCs was not significantly correlated with enhancement in the FCs in ASD (that is, correlation between the distance of an FC and its *r*_ASD_−*r*_TD_; difference in correlations of FCs for ASD and TD, *r*=0.42, *P*=0.10) or with the difference in their absolute strengths (|*r*_ASD_|−|*r*_TD_|; *r*=0.24, *P*=0.36). Furthermore, there was no significant difference (*t*_14_=1.3, *P*=0.23) between the mean distance of nine FCs exhibiting under-connectivity (64.6±51.1 mm) and the mean distance of seven FCs exhibiting over-connectivity (92.8±33.9 mm). Here the distance of each FC was computed as a one-line distance between the central coordinates of the two connected brain regions.

### Prediction of diagnostic instrument scores using the 16 FCs

**Figure 4:**
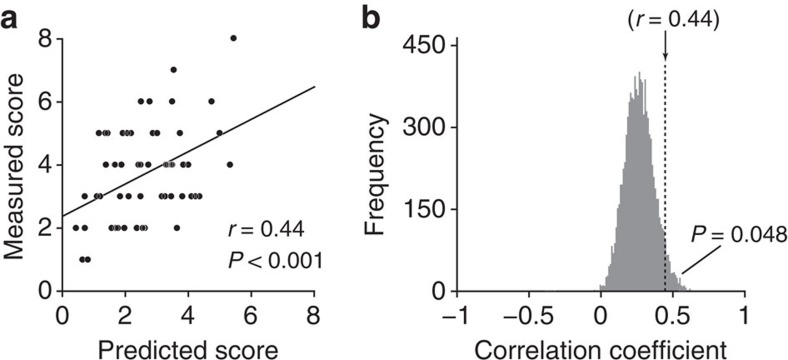
Prediction of ADOS A domain score (communication) using the 16 FCs identified in the classifier. (**a**) Scatter plot of the measured ADOS A domain versus the predicted score, which was computed as a linear weighted summation of the 16 FCs identified by the ASD/TD classifier. Each dot represents individual data (*n*=58, see Methods, Participants). The line indicates the linear regression of the measured score from the predicted score, and correlation coefficient and statistical significance are shown (see Supplementary Table 3 for results of the other three domains of ADOS and all four domains of the ADI-R instrument). (**b**) The frequency of the different correlation coefficient values is plotted in a bootstrap analysis in which 16 FCs were randomly selected from all 9,730 FCs, with the exception of those 42 FCs selected in the LOOCV procedure. The correlation coefficient between the measured and predicted scores was computed as in **a**. This analysis indicates that the probability of obtaining the correlation coefficient *r*=0.44 was small (*P*=0.048), and demonstrates that the 16 FCs identified in the classifier specifically contain information useful to predict the ADOS A score.

Using the 16 FCs identified in the classifier, we predicted the measured domain scores of the two standard diagnostic instruments, the Autism Diagnostic Observation Schedule (ADOS)^47^ and the Autism Diagnostic Interview-Revised (ADI-R)^48^. The number of available subjects was 58 for ADOS and 27 for ADI-R. Each instrument contained four domains, as summarized in Supplementary Table 3. In each domain of each instrument, a linear regression was individually employed to determine the weights of 16 FCs so that their weighted linear summation was used as a predictor for the corresponding measured score. Among the total of eight domains of ADOS and ADI-R, we found that the communication domain of the ADOS (ADOS A) was well predicted from the 16 FCs with statistically significant correlation (*r*=0.44, uncorrected *P*=0.001<0.05/8, a Bonferroni-corrected threshold for multiple comparisons; see Fig. 4a and Supplementary Table 3). The bootstrapping analysis demonstrated that the probability that this correlation *r*=0.44 would be derived from 16 FCs randomly selected from 9,688 (=9,730−42) FCs, which were not identified in the LOOCV procedure, was small (*P*=0.048, Fig. 4b, see also Methods). These results demonstrate that the 16 FCs identified in the classifier specifically contain more useful information than the remaining FCs in predicting the ADOS A score, which is the degree of deficits in communicative behaviours.

### Application of the ASD classifier to other disorders

Here we examine the specificity to ASD of the ASD classifier, and/or its generalizability to psychiatric disorders other than ASD. If the ASD classifier predicts patients with a different disorder as healthy control individuals, then AUC by the ASD classifier for the classification of patients with that disorder from their control should be close to 0.5. In this case, we may as well say that the patients possess so little ASD-ness and that the disorder is not related to ASD from the viewpoint of the imaging biological dimension. In contrast, if the ASD classifier perfectly discriminates patients with a different disorder from its control, the classification AUC should be close to 1. In this case, we may as well say that the patients possess a large degree of ASD-ness in them and that this disorder is closely related to ASD according to the biological dimension.

**Figure 5:**
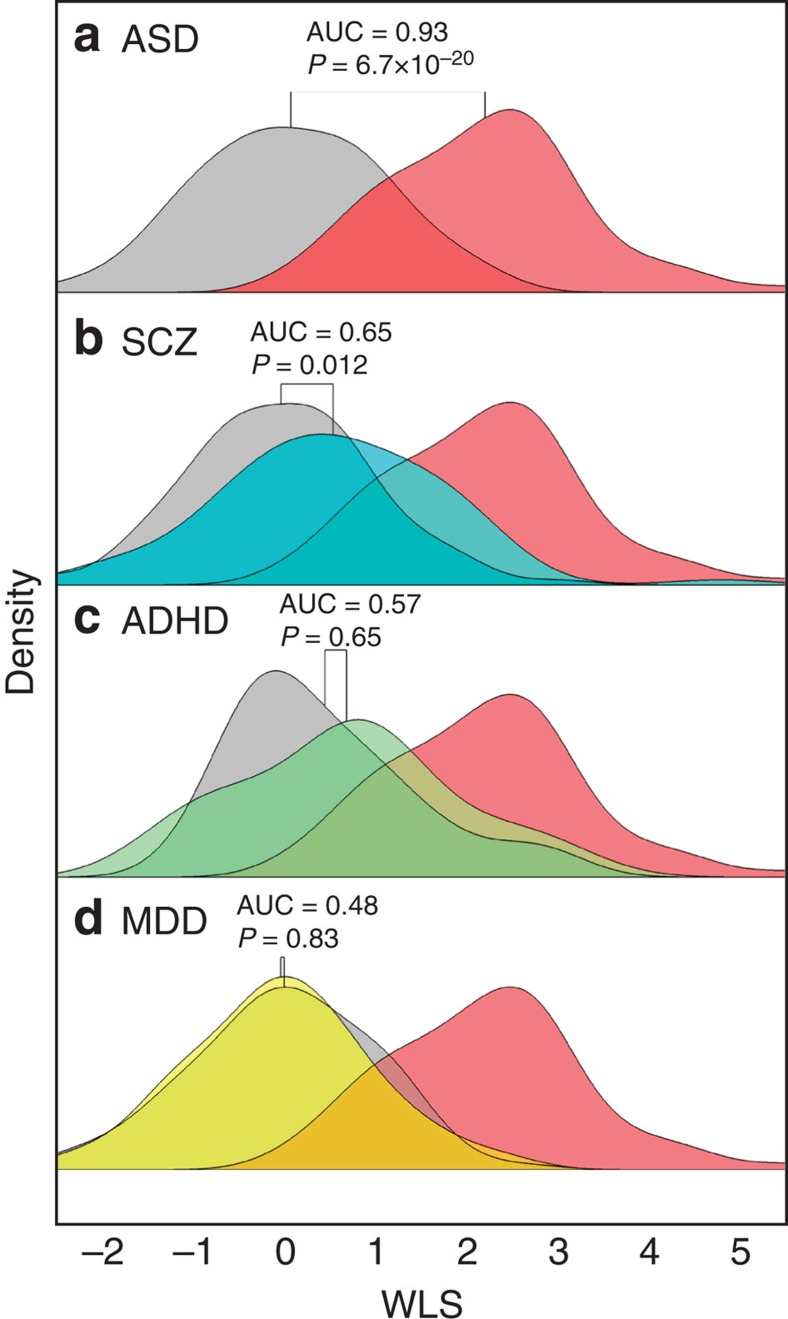
Application of the ASD classifier to other psychiatric disorders. The density distributions of the weighted linear sum (WLS) obtained by applying the ASD classifier to (**a**) ASD, (**b**) SCZ, (**c**) ADHD and (**d**) MDD data sets. In each panel, the patient distribution and the TD/healthy control distribution are plotted separately, with coloured and grey areas, respectively. For reference, the WLS distribution of the ASD patients (red area) in **a** is duplicated across the panels (**b**–**d**). For each patient–control pair in **a**–**d**, the significance of the Benjamini–Hochberg-corrected Kolmogorov–Smirnov test and AUC values are shown. In this figure, for the visualization purposes, the WLS of each data set is standardized to match median and s.d. of TD controls across the panels. Note that this WLS standardization is not performed in any quantitative analysis.

To test this, we applied the ASD classifier to two additional Japanese cohorts of SCZ and MDD (each containing a healthy control population) and one European cohort of ADHD (containing a TD population) (see Methods). We computed the WLS of the identified FCs in the ASD classifier, that is, the ASD-ness of each individual within the SCZ, MDD and ADHD data sets, and their corresponding healthy or TD control populations. We then compared the WLS distributions between each disorder group and its corresponding healthy or TD control (Fig. 5). As expected and already demonstrated in Fig. 1, the separation of WLS distributions was the largest between ASD and TD (Fig. 5a), meaning that the developed ASD classifier has a good ability to discriminate ASD from TD individuals, and the ASD-ness is able to successfully separate the two populations. The separation between SCZ individuals and their healthy controls was poorer than that of ASD but statistically significant (Fig. 5b; AUC=0.65, Kolmogorov–Smirnov test, *P*=0.012 corrected for multiple comparisons). In contrast to SCZ, the WLS distributions of ADHD and MDD, and their corresponding TD and healthy controls were not distinguishable (Fig. 5c,d; AUC=0.57, Kolmogorov–Smirnov test, *P*=0.65 for ADHD; AUC=0.48, Kolmogorov–Smirnov test, *P*=0.83 for MDD). In other words, the ASD-ness of individuals with ADHD or MDD is not different from that of their controls. Note that MDD was more completely indistinguishable from its control compared with ADHD according to the ASD-ness. It can be said that the ASD classifier was specific to ASD regarding ADHD and MDD, but was modestly generalized to SCZ (compare AUC=0.93 for the ASD discovery cohort and AUC=0.65 for the SCZ data set). These results demonstrate that the WLS of the ASD classifier, in other words the ASD-ness, quantified the spectrum of the four disorders as follows; SCZ was close to ASD, ADHD was distant from ASD, and MDD was farthest from ASD.

## Discussion

In the present study, we established a reliable neuroimaging-based classifier for ASD by investigating the whole-brain patterns of FCs using the rs-fcMRI data of 74 adults with ASD and 107 TD individuals collected at multiple sites in Japan. This classifier incorporated as small as 16 FCs (only 0.2% of the entire FCs) distributed across the brain but not contained in the left hemisphere, and allowed a diagnosis prediction accuracy of 85% for each individual with balanced sensitivity and specificity of 80% and 89%, respectively. Most importantly, the high performance of the classifier was generalized across the Pacific to the independent, ethnically more diverse, validation cohort in the USA (75% accuracy) with only 10% decrease in accuracy compared with the Japanese discovery cohort. Although successful construction of a FC-based ASD classifier has been reported previously^49^, our work presents the first achievement of successful classification for ASD across discovery as well as validation cohorts. Our approach may lay a foundation for developing classifiers for other psychiatric disorders. This is because, to the best of our knowledge, there exists no neuroimaging-based classifier for any psychiatric disorder of which the generalization capability is demonstrated for an independent validation cohort. We have further applied the ASD classifier for classification of SCZ, MDD and ADHD patients from their healthy controls. The results indicate that, while the ASD classifier exhibited hardly any generalizability as applicable to ADHD and MDD, it can be to a modest degree generalizable to SCZ.

In the present study, both sophisticated machine-learning algorithms and a variety of data from the Japanese three sites were essential for successful generalization of the classifier across multiple sites. First, the unique combination of the two machine-learning algorithms, *L*_1_-SCCA and SLR, worked to achieve optimal extraction of a small number of FCs that were relevant only to the core ASD characteristics. This optimal extraction avoids over-fitting and eliminates the effects of NVs such as age, sex and site-dependent characteristics in the data composition and imaging protocols. In fact, when we applied to our data sets a state-of-the-art machine-learning algorithm of nested cross-validation of the elastic net^50^, this algorithm selected 10 times more FCs and performed 14% worse on the USA cohort (percent correct of 61%; see Supplementary Note 3 for more detail). This algorithm did not explicitly exclude the interfering effects of NVs. This fact indicates that the generalization capability of our ASD classifier across imaging sites most probably stemmed from reduced influences of FCs related to NVs owing to the unique combination of *L*_1_-SCCA and SLR. By utilizing synthetic data sets and by comparing with elastic net, we demonstrated that the unique combination of *L*_1_-SCCA and SLR can be useful to reduce the influence of FCs related to NVs (see Supplementary Note 4). Second, we examined the conditions for which data sets can derive reliable ASD classifiers. More concretely, we selected one or two of the three sites within the Japanese discovery cohort for training data sets (Supplementary Table 4) and constructed classifiers. In addition, we trained a classifier with the ABIDE data set and examined its generalization capability for the Japanese cohort (Supplementary Note 5). The resulting performances were generally poorer than that of the current ASD classifier. Especially, the performance of the ASD classifier, which was developed using the ABIDE data set, was very poor. It is suggested that having a sufficient number of participants in total as well as in each site is a necessary condition to construct a reliable ASD classifier (see also Methods). We conclude that both a sophisticated machine learning algorithm and a large training data set are essential for developing a reliable and generalizable classifier.

It is worth noting that the performance of the classifier may be upper-limited by the aetiological and phenotypic heterogeneity of ASD, which is likely to be accompanied by differential biological underpinnings. This limitation may be ameliorated and the overall diagnostic precision may be improved by identifying subgroups that are biologically more uniform within a given population and by extracting a set of features that characterize each subgroup.

What do the results of the applications of the ASD classifier to the other psychiatric disorders suggest? Figure 5 shows the density distributions of the WLS that resulted from the application of the ASD classifier to data sets for other disorders, SCZ, MDD and ADHD. The results raise the intriguing possibility that the degree of generalizability and specificity of the ASD classifier to these other disorders reflect their spectral structure on the scale of whole-brain intrinsic functional networks. From this perspective, Fig. 5 suggests that ASD shares more intrinsic-functional networks with SCZ than with ADHD or MDD. This is consistent with the results of the previous clinical works as described in the opening paragraphs^30^,^32^. These results raise the possibility that a neuropsychiatric disorder can be redefined and represented as a location in a multi-dimensional space defined by FC-based biological ‘dimensions', each of which takes the form of WLS consisting of a small number of FCs^34^,^35^. In this case, the ‘ASD-ness' could make a dimension along which ASD and SCZ are located and might be orthogonal to another dimension along which ADHD and MDD are located. Interestingly, to aim at building a biomarker for ASD, we started with a categorical approach by which a supervised machine-learning algorithm was utilized and a diagnostic label was adopted as its teaching signal. However, the results of applying the classifier built for that original purpose to other neuropsychiatric diseases have raised the exciting possibility that the classifier allowed us to go beyond the category regime and to embark on the exploration for new biological dimensions^34^,^35^.

How would the current research results contribute to the future diagnoses and therapy for neuropsychiatric disorders? Recently, an increasing number of researchers have had the perspective that a biomarker can be used to stratify a broad illness phenotype into a finite number of treatment-relevant subgroups, thereby bypassing nosological arguments over diagnostic boundaries^5^. This perspective could lead to developments of multiple neuroimaging-based biomarkers for multiple psychiatric disorders. The generalization capability across imaging sites is a bare minimal requirement for a classifier towards its clinical applications. If reliable classifiers for ASD, SCZ, MDD, ADHD and even other disorders are developed in the near future, these classifiers may be utilized as a new diagnostic tool as a set of biomarkers that can quantify intrinsic FCs of individuals who need in-depth clinical examinations for various disorders. Such quantification is possible because multiple biomarkers can use the same rs-fcMRI data from an individual. In this vein, the current study could provide a foundation for such a future direction in neuropsychiatric diagnoses.

We formed a consortium with other researchers in 2013 as part of the Japanese Strategic Research Program for Promotion of Brain Science (SRPBS)^51^ with the aim of applying big data, machine-learning algorithms and sophisticated fMRI neurofeedback methods^52^,^53^ to study diagnosis and therapy for multiple psychiatric disorders. We have developed fMRI real-time neurofeedback methods that can change FC between brain regions with healthy and ASD individuals. First, in healthy individuals, 4 days of fMRI real-time neurofeedback of FC between two designated areas changed rs-fcMRI connectivity, and the changes remained for more than 2 months^53^. Second, for ASD individuals within the SRPBS consortium, a further developed neurofeedback method of FC has been applied^54^,^55^. The identified 16 FCs in the present research may contribute to improvements in the fMRI real-time neurofeedback of FC to ASD individuals as a possible therapeutic target. In one recent study with ASD individuals, target FCs were selected by the ASD classifier developed in the present study^54^, and the WLS of the ASD classifier was estimated in a real-time fMRI neurofeedback paradigm for an ASD individual. Then, a sign-inverted WLS value was presented to the ASD individual as a neurofeedback target to increase in a reinforcement learning paradigm^54^. Increases in the neurofeedback score lead to reductions in the WLS value. Researches within the SRPBS consortium have suggested that the ASD classifier we have developed in this study could be a useful tool, with which connectivity neurofeedback methods would make further progress to attain the goal of ASD individuals obtaining normal rs-fcMRI dynamics^51^.

In summary, we have developed a generalizable rs-fcMRI-based classifier for ASD^28^ for the first time. Despite the fact that this classifier is based on a small number of identified FCs, it greatly distinguishes ASD from TD with demonstrated generalization in the independent validation cohorts, and accounts for socio-communicative aspects of ASD. The results of applications of this ASD classifier to other psychiatric disorders have left open the interesting possibility of exploring new neuroimaging-based dimensions for multiple-disorder spectrum.

## Methods

### Participants

All participants in the present study provided written informed consent as approved by the ethics committees of the recruiting institutions as follows: the Ethics Committee of the Graduate School of Medicine and Faculty of Medicine at the University of Tokyo, the Ethics Committee of the Faculty of Medicine of Showa University, the Institutional Review Board of Advanced Telecommunications Research Institute International, the Committee on Medical Ethics of Kyoto University and the Ethics Committee of Hiroshima University.

A total of 74 adults with ASD and 107 age, sex, handedness and IQ-matched TD individuals participated in the present study. The participants were recruited at three different sites in Japan (sites A–C). Their demographic information is summarized in Supplementary Table 1.

At site A, participants with ASD were recruited through the Department of Child Psychiatry and Neuropsychiatry at the University of Tokyo Hospital and via an advertisement on the website of the University of Tokyo Hospital. All ASD participants (*n*=35) were diagnosed with pervasive developmental disorder (PDD) based on the DSM-IV-TR criteria^56^. DSM-IV-TR diagnoses of autistic disorder, Asperger's disorder, or pervasive developmental disorder not otherwise specified (PDD-NOS) (*n*=24, *n*=3 and *n*=8, respectively) were supported by ADOS^47^ (*n*=35) and ADI-R^48^ (*n*=27). The Japanese version of mini-international neuropsychiatric interview M.I.N.I. was used to evaluate psychiatric comorbidity^57^. No participant satisfied the diagnostic criteria for substance use disorder, bipolar disorder or SCZ. The IQ scores of participants with ASD were obtained using the Wechsler adult intelligence scale-revised (WAIS-R) or third edition (WAIS-III). The full-scale IQs (FIQs) of all of the individuals with ASD were measured and found to be >85. TD individuals were recruited from the local community and via other on-going studies at site A. M.I.N.I. was used to confirm that none of the TD met the diagnostic criteria for any psychiatric disorder. The IQs of the TD individuals were estimated using the Japanese version of the national adult reading test (JART)^58^. All participants were right-handed according to the Edinburgh Handedness Inventory^59^. Participants completed the Japanese version of the autism-spectrum quotient (AQ-J)^60^. At the time of scanning, 11 ASD individuals were medication free, whereas the remaining 24 ASD individuals had been administered the following psychotropic drugs: anxiolytics (*n*=18), antidepressants (*n*=20), antipsychotics (*n*=15), antiepileptics (*n*=6) and sleep-inducing drugs (*n*=17), before the scanning. Some participants had been administered multiple drugs (*n*=20).

At site B (Showa University Karasuyama Hospital), the diagnoses were made by a team of experienced psychiatrists and clinical psychologists based on detailed interviews of individuals regarding their development and behaviour, from infancy through adolescence and family history. At least one caregiver who knew the individual in his/her childhood was usually present in the interview. At the end of the clinical interview, the psychiatrist diagnosed the individuals according to the DSM-IV-TR diagnostic criteria for PDD based on the consensus of the psychiatrists and the clinical psychologists. These assessments resulted in all of the participants in the ASD group (*n*=39) receiving clinical diagnoses of autistic disorder (*n*=19), Asperger's disorder (*n*=16) and PDD-NOS (*n*=4). The diagnoses for 23 individuals were supported by ADOS^47^. As performed at site A, the IQ scores of participants with ASD were obtained using WAIS-R or WAIS-III. FIQ for all individuals with ASD was >80. FIQ for all TD individuals was estimated using JART. All participants, including ASD and TD, completed the Japanese version of the AQ test^60^. M.I.N.I. was used to confirm that none of the TD individuals met the diagnostic criteria for any psychiatric disorder. At the time of scanning, 27 ASD individuals were medication free, whereas the remaining 12 ASD individuals were administered the following psychotropic drugs: anxiolytics (*n*=7), antidepressant (*n*=9), antipsychotics (*n*=3), antiepileptics (*n*=3) and sleep-inducing drugs (*n*=7). Some participants were administered multiple drugs (*n*=8).

At site C (Advanced Telecommunications Research Institute International), 33 TDs participated in the present study after providing written informed consent. None of the participants had a previous history of neurological disorders. All participants were right-handed as confirmed by the Edinburgh inventory. No ASD individuals were recruited at site C as it was not a medical institution.

### Training data set used for construction of the ASD classifier

MRI data for the training of the ASD/TD classifier were acquired at three different sites in Japan. An MRI system was used at the site where participants were recruited. Each imaging site adopted its own imaging protocol (Supplementary Table 5), differing in both imaging parameters and instructions provided to the participants during the scan. These discrepancies were taken into account in subsequent classification analysis (see the section below ‘*L*_1_-regularized sparse canonical correlation analysis' and Supplementary Note 6). At all sites, participants were subjected to high-resolution *T*_1_-weighted structural imaging as well as resting-state functional imaging, which were preprocessed with SPM8 (Wellcome Trust Centre for Neuroimaging, University College London, UK) software running on MATLAB (R2012b, Mathworks, USA) as follows. First, the raw functional images were corrected for slice-timing and realigned to the mean image of that sequence to compensate for head motion. Next, the structural image was co-registered to the mean functional image and segmented into three tissue classes in the Montreal Neurological Institute (MNI) space. Using associated parameters, the functional images were then normalized and resampled in a 2 × 2 × 2 mm^3^ grid. Finally, they were smoothed by Gaussian of full-width at half-maximum 6 mm. Because subsequent analysis is based on the pattern of temporal correlations among the brain regions and the evaluation has shown itself to be highly sensitive to abrupt head motion during scanning, the pre-processed sequence of functional images was examined as follows. First, we evaluated the mean relative displacement^61^ in each of the six motion parameters (that is, translation along and rotation with respect to the *x*, *y* and *z* axes) by calculating the mean of the absolute frame-to-frame relative changes in each individual parameter through a given time series (namely, the mean of 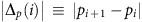
across the time series, where *p* is one of the six motion parameters and *i* specifies the time point). In both the Japanese and the USA data sets, there was no statistically significant group difference in this measure for any of the six motion parameters (Supplementary Table 6). Next, for each participant, we calculated the frame displacement (FD) at each time point by summing 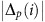 for all six parameters. Using this FD, we employed the ‘scrubbing' procedure^62^ to identify and exclude any frame exhibiting excessive head motions. Specifically, a frame was flagged and removed, along with the previous and two subsequent frames, from the correlation analysis, if the associated FD exceeded 0.5 mm (ref. 62). On average (±s.d.), 93.4±11.2% and 95.7±7.5% of the original frames passed this ‘scrubbing' procedure in the TD and ASD populations, respectively, and these fractions did not differ significantly between the two populations (two-sample *t*-test, *P*=0.13).

### Generalization to USA data

The performance of the classifier was further tested using an independent data set available from the US ABIDE Project^40^. For individuals with ASD, the criteria for data inclusion were that they must (1) be older than 18 years of age, (2) be right-handed, (3) have a FIQ exceeding 80, (4) have no comorbidity and (5) have been diagnosed as autistic by either ADI-R or ADOS. Individuals with an explicit medication record at the time of the scan were excluded. A total of 54 autistic individuals satisfied these criteria. In addition, 52 age-, sex- and FIQ-matched TD individuals satisfying criteria (1)–(4) were selected from the same pool of participants. Then, their MRI data were obtained and the functional images were visually inspected to ascertain that the field of view covered the entire brain. We found that functional images for 18 participants suffered from severe truncation at either the top (parietal lobe) or bottom (inferior temporal lobe to cerebellum) portion of the brain, and they were removed from the further analysis accordingly. A total of 44 individuals with ASD and 44 demographically matched TD individuals comprised the final list. Their properties are summarized in Supplementary Table 7. The imaging protocols adopted at each site are summarized in Supplementary Table 8. We preprocessed the MRI data in the same way as for the Japanese data and calculated the interregional FC for each subject. In addition, to test the performance of the classifier on individuals with more diverse profiles, we formed a Supplementary Dataset by removing the conditions for FIQ, comorbidity and medication status. The detail of the data set and the result of the analysis are provided in Supplementary Note 2.

### Further evaluation of the generalization capability

To further examine the classifier's generalization capability, we formed an extra data set at site B (Showa University Karasuyama Hospital) independently of the training data set. This data set incorporated 27 individuals with ASD and 27 demographically matched TDs who were recruited under the criteria of this site (see the Participants section). The data acquisition was conducted using a new 3 T MR scanner of a different manufacturer, replaced after the formation of the training data set. For more detail including the imaging protocol, see Supplementary Table 9.

### Application of the ASD classifier to other disorder

To further understand the generalization property of the ASD classifier, we evaluated its classification specificity to ASD. Specifically, we tested its classification performance using the data sets that incorporated individuals with SCZ, MDD and ADHD as follows.

We formed a data set that consisted of 66 patients with SCZ (34 females, age 38.2±9.1 year) and 107 age-matched healthy control participants (40 females, age 34.6±8.2 year). Their handedness was determined by the Edinburgh Handedness Inventory^59^. The mean±s.d. of the SCZ was 75.0±40.9 and that of the healthy control was 81.2±35.1. The patients were recruited at in- and out-patient facilities in the Kansai region in Japan. They were diagnosed with the patient edition of the Structured Clinical Interview for DSM-IV Axis I Disorders (SCID). None of the patients had comorbid psychiatric disorders. The controls were recruited from the local community at Kyoto University. None of them had any history of psychiatric illness, as indicated by screening results using the nonpatient edition of the SCID. These screening results were confirmed by the fact that none of their first-degree relatives had any history of psychotic disorders. The MR data of both patients and healthy controls were acquired at the Kyoto University Hospital (see Supplementary Table 9 for more details). All participants were physically healthy when they were scanned. The details of this data set are stated elsewhere.

Next, we formed a data set that consisted of 105 patients with unipolar MDD (51 females; 42.8±11.5 year) and 145 age-matched healthy control participants (90 females; 39.5±12.7 year). Their handedness was determined by the Edinburgh Handedness Inventory^59^. The mean±s.d. of the MDD were 84.8±30.1 and that of the healthy control populations was 81.7±36.0. The patients were recruited from a local clinic and the healthy controls in the community of the Hiroshima University. The MR data of both patients and healthy controls were acquired at the Hiroshima University Hospital and other local imaging facilities (see Supplementary Table 9 for the summary of the data). All the patients were screened with the DSM-IV criteria for a unipolar MDD diagnosis using M.I.N.I. No patient had current or past SCZ episodes. Healthy participants were interviewed with M.I.N.I. and none of them showed a history of psychiatric disorders according to DSM-IV criteria. The details of this data set will be described elsewhere.

Next, we formed a data set that incorporated individuals with ADHD and TDs acquired by the NeuroIMAGE project in the Netherlands (http://www.neuroimage.nl/). They were a part of the ADHD-200 Sample and we obtained their permission-free MR data and the associated demographic and phenotypic information from the ADHD-200 Sample website (http://fcon_1000.projects.nitrc.org/indi/adhd200/) under an unrestricted usage agreement for non-commercial research purposes. Because the current study focuses on adult ASD, only individuals with rounded ages >18 years were incorporated into the present analysis. The final data set consisted of 13 individuals with ADHD (2 females; 19.0±1.1 year) and age-matched 13 TDs (7 females; 19.2±1.2 year). The subtype identification of the ADHD populations was three hyperactive-impulsive types, one inattentive type and nine combined (hyperactive-impulsive and inattentive) types. Among the ADHD individuals, 10 were right-handed, 3 were left-handed and no information was available for 1 individual. Among the TD individuals, 11 were right-handed and 2 were left-handed. For more information on their demographic and phenotypic properties, see the ADHD-200 Sample website.

To evaluate the extent to which each additional disorder tends to share traits with ASD, we applied the ASD classifier to these three data sets in the same manner as the US ABIDE data. The AUC of the classification was computed to evaluate the degree of separation of SCZ, MDD and ADHD from their corresponding control population. Moreover, the Kolmogorov–Smirnov test was used to compare the WLS distributions of each disorder with their corresponding healthy controls and TD individuals. The WLS distributions of the patient and control populations were fitted separately with a mixture of Gaussians distributions and illustrated in Fig. 5, along with the respective AUC and Kolmogorov–Smirnov *P* value. The *P* value of each test was corrected for multiple comparisons by the Benjamini–Hochberg procedure. For visualization purposes, the WLS in each data set was standardized such that the median and s.d. of healthy controls and TD individuals were matched across panels. It should be noted that this standardization was not used for any statistical analyses.

### Interregional correlation analysis

For each participant, a pair-wise, interregional FC was evaluated among 140 regions of interest (ROIs) covering the entire brain. The spatial extent of each region was defined anatomically by the digital atlas of the Brainvisa Sulci Atlas (BSA)^36^. Because this atlas did not include the cerebellum, the subregions of the cerebellum were appended to it by incorporating their boundary definitions in the anatomical automatic labelling (AAL) package^63^. Although the original AAL atlas defined 26 subregions in the cerebellum, they were reorganized into the following three subregions in this study: the left and right cerebellum, and the vermis. This modification was necessary because the scanned volume did not cover the entire cerebellar regions for some individuals, and this incomplete coverage lead to missing elements in the correlation matrix. To surmount this, the mean time courses in the cerebellar regions were evaluated in three broader subregions by masking, if any, unavailable voxels in each individual. This BSA-AAL composite atlas was resampled in the 2 × 2 × 2 mm^3^ grid MNI space. The representative time course in each region was extracted by averaging the time courses of the voxels therein. A band-pass filter (transmission range, 0.008–0.1 Hz) was applied to these sets of time courses prior to the following regression procedure. The filtered time courses were linearly regressed by the temporal fluctuations of the white matter, the cerebrospinal fluid, and the entire brain as well as six head motion parameters. Here, the fluctuation in each tissue class was determined from the average time course of the voxels within a mask created by the segmentation procedure of the *T*_1_ image. The mask for the white matter was eroded by one voxel to consider a partial volume effect. These extracted times course were bandpass filtered (transmission range, 0.008–0.1 Hz) before the linear regression, as was done for regional time courses. Then, for each individual, a matrix of 9,730 FCs between 140 ROIs was calculated by exhaustively evaluating pair-wise temporal Pearson correlations of blood oxygenation level dependent signals time courses while discarding flagged frames, if any, in the previous procedure (scrubbing). We note that unfiltered motion-related regressors, such as six head motion parameters in the present case, could reintroduce high-frequency fluctuations into the time course data^61^. The scrubbing procedure was employed to remove any frames exhibiting abrupt head motions that could be the source of high-frequency fluctuation in the filtered time course^64^. In addition, we confirmed that there was no statistically significant difference in any of the motion parameters between the ASD and TD populations (Supplementary Table 6). Thus, the chance that the classification was influenced by head motion remained minimal.

### Selecting FCs as the ASD classifier

Two major challenges exist in constructing a classifier for ASD. The first challenge is the problem of over-fitting, because of the small sample size. As previously mentioned, the dimension of the input to the classifier is *M*=9,730. However, the amount of data is only *N*=181. Because *N* is much smaller than the dimension of data *M*, the parameters of the classifier can be easily over-fitted to the training data. Because of this over-fitting, the constructed classifier will likely exhibit extremely poor performance with newly sampled test data, which are not used in training the classifier. Therefore, we need to properly introduce regularization to identify and utilize only essential FCs to ensure good generalization of the classifier. Here we adopted a cascade of the *L*_1_-regularization method, a well-known approach for managing the problem of small sample size, and a sparse estimation method with automatic relevance determination^65^,^66^, as detailed below and in the next two sections.

The second major challenge is related to NVs and is due to the fact that an ASD classifier is clinically useful and scientifically trustworthy only if it maintains good performance for MRI data scanned at imaging sites different from the sites where the training data were collected. This is the so-called generalization capability across imaging sites. However, in clinical applications, it has often been observed that a classifier trained using data acquired from a particular site cannot be generalized to the data scanned at other sites^7^,^8^,^26^. We overcame the second challenge by using a wide variety of training data sets obtained at the three imaging sites, and by the unique combination of two sophisticated machine-learning algorithms: *L*_1_-SCCA and SLR. Through additional analyses, we confirmed that MRI data scanned from at least three sites, and obtained under a variety of imaging conditions, are necessary to train a classifier that generalizes across multiple sites (see Supplementary Table 4 for the results obtained when data from only one or two sites were used). Furthermore, to extract FCs essential for ASD classification and to reduce undesirable effects due to different scanning conditions and demographic distributions at different sites, that is, irrelevant NVs, we adopted the *L*_1_-regularized sparse canonical correlation analysis (*L*_1_-SCCA)^37^ (see also Supplementary Note 6).

Altogether, the procedure for selecting relevant FCs, training a predictive model and assessing its generalization ability was carried out as a sequential process of 9 × 9 nested feature-selection and leave-one-out cross-validation (see also the schematic diagram in Supplementary Fig. 2). In each leave-one-out (LOO) cross-validation (CV) fold, all-but-one subjects were used to train a SLR^38^ classifier, while the remaining subject was used for evaluation. SLR has the ability to train a logistic regression model, while objectively pruning FCs that are not useful for the purpose of classifying ASD. Before training SLR, it is necessary to reduce the input dimension to some extent and simultaneously remove the effects of NVs that may cause catastrophic over-fitting. Therefore, prior to LOOCV, nested FS was performed using *L*_1_-SCCA. *L*_1_-SCCA identifies the latent relationships between FCs and various attributes of each individual, including the diagnostic label, available demographic information and imaging conditions (see details in the next section). By selecting FCs that have a connection with a canonical variable related only to the ‘Diagnosis' label and not to NVs, we aimed to reduce the interferential effects of NVs.

The feature (FCs) selection procedure was similar to 9 × 9 nested cross-validation, with the difference being that the test set was never used for validation or feature (FCs) selection (Supplementary Fig. 2). In this way, *L*_1_-SCCA was trained on different subsamples of the data set, to increase the stability of the selected features. The ‘test set' of the outer loop FS process was kept as a testing pool for LOOCV, whereas the nine folds of the inner loop FS were used to select features. Consequently, the LOOCV folds that belonged to the same testing pool of the outer loop FS shared the same reduced features. In the inner loop FS, the *L*_1_-SCCA hyperparameters *λ*_1_ and *λ*_2_ were varied independently between 0.1 and 0.9 (*λ*_1_≤*λ*_2_) with a step of 0.1. For each instance of *L*_1_-SCCA, we found the canonical variables connected only with the label ‘Diagnosis' and kept the features associated with those canonical variables. On average, the number of *λ* combinations that complied with this constraint was 17.6±5.0% of the total 45 possible combinations. The features selected at each inner fold and *λ* combination were combined by the union operation, to include features that are important for any possible subsample (inner nine folds) of the training data set. This procedure leads to the selection of 4,529±161 FCs (mean and s.d. across outer folds). Once the inner loop FS was executed, one sample was taken from the testing pool of the outer loop FS, and used as the test set of the LOOCV. The remaining samples were used to train SLR on the FCs retained during the inner loop FS. The advantage of this nested FS procedure was that it used most of the data to train the models (LOOCV), while performing time-efficient FS (9 × 9 nested FS). The FS procedure based on *L*_1_-SCCA is time consuming, and doing it for each fold of the LOOCV is not computationally feasible. Instead, we kept one of the nine outer folds as a ‘testing pool' for the LOOCV and ran *L*_1_-SCCA on the remaining eight folds. This means that we could reuse the selected features for all the samples in the ‘testing pool', during the LOOCV. That is, by performing ninefold nested FS we kept the test set of the LOOCV separate from the data set used to select features. We could efficiently (from a computational time perspective) avoid information leakage and over-optimistic results^20^. It should be noted that, to split the data into nine folds, we used a stratified approach, so as to keep an equal amount of (diagnosis, gender and site) combinations per fold.

One of the advantages of the proposed algorithm is that it does not rely on parameter tuning. Indeed, the *L*_1_-SCCA procedure is inspired by the ‘stability-selection^67^' approach, where subsampling is combined with selection algorithms. Specifically, it was designed in such a way that the amount of regularization is not chosen explicitly based on the *L*_1_-penalty tuning, but rather on concatenation of the features selected by different *L*_1_-penalties and subsamples. In the original ‘stability-selection' paradigm, features are selected based on the frequency of selection across subsampling repetitions, which typically requires threshold tuning by additional cross-validation. In this study, the union of the FCs selected by *L*_1_-SCCA on different subsamples aims to avoid the tweaking of such additional parameters. Moreover, SLR relies on automatic relevance determination, a Bayesian procedure that does not require parameter tuning.

To classify the independent cohort data sets (for example, the US ABIDE data set), we trained the final SLR classifier based on the union of the features selected throughout the 9 × 9 nested FS, using all the Japanese subjects as the training set.

### *L*_1_-regularized sparse canonical correlation analysis

In general, by employing canonical correlation analysis (CCA)^68^, we can identify latent relationship between paired observations. Specifically, CCA can derive projection vectors so that the paired projected variables (called canonical variables) have maximum correlation. Suppose that we have *N* observations of the paired variables 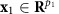 and 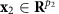. Let 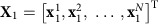 denote the *N* × *p*_1_ matrix comprising the first set of variables, and let 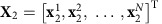 denote the *N* × *p*_2_ matrix comprising the second set of variables. As we explained in the previous section, we use sparse CCA with *L*_1_-norm regularization, *L*_1_-SCCA^37^. We assume that the columns listing **X**_1_ and **X**_2_ of the training set have been centred to have zero mean and scaled to have unit variance. For one canonical variable, *L*_1_-SCCA can then be formulated as


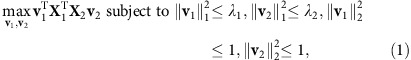


where hyperparameters *λ*_1_ and *λ*_2_ indicate the sparseness of the projection vectors **v**_1_ and **v**_2_, respectively. Since the maximum number of canonical variables is *q*=min(*p*_1_, *p*_2_), the projection matrices are defined as 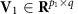 and 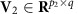, where each column contains the projection vector that is associated with a canonical variable.

To identify the latent relationships between demographic information and FC, we constructed two data matrices. A row of the first data matrix **X**_1_ lists the properties and attributes of a subject, including the diagnosis (ASD or TD), site information indicating where the brain activities of the subject were scanned, age, sex, imaging conditions (open or closed eyes) and status of medication (antipsychotics, antidepressant and anxiolytics, separately) (see Supplementary Fig. 7). More specifically, the number of columns of the demographic information data matrix **X**_1_ is 10, that is, *p*_1_=10. The first column contains either 1 (=ASD) or 0 (=TD). The next three columns contain either [1 0 0] (=site A), [0 1 0] (=site B) or [0 0 1] (=site C). The fifth column contains age value, the sixth column contains either 1 (=male) or 0 (=female), the seventh column contains either 1 (=eye open) or 0 (=eye closed) and the last three columns contain status of the three medications, where each column contains either 1 (=with medication) or 0 (=without medication). The second data matrix **X**_2_ pools a row-vector form of the off-diagonal lower triangular portion of a correlation matrix that represents the FC of a subject. *L*_1_-SCCA was applied to the pair of matrices **X**_1_ and **X**_2_, from which the sparse projection matrices **V**_1_ and **V**_2_ were derived. In this study, we defined the constraint that at least one canonical variable should be associated only with the diagnostic label, and we called it diagnostic canonical constraint. In addition, we call canonical variables that are associated only with the diagnostic label as diagnostic canonical variables. This was achieved by looking at the columns of **V**_1_ that had a non-zero element only in the first row (that corresponds to the diagnostic label). Subsequently, only the columns of **V**_2_ corresponding to the diagnostic canonical variables were used to form the vector 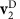, by computing the sum of the absolute value across columns (that is, union of features across ‘diagnostic canonical variables'). Moreover, we obtained the union of features (that is, 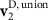) across repetitions of the inner loop FS and across 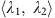 meeting diagnostic canonical constraint, by computing the sum of all the 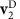 derived in the process. Then, we used 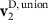 to identify the indices of the connectivity vector relevant to the diagnosis label. We projected the original connectivity vector into a subspace defined by the nonzero elements of 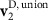. Here we defined a variable *i*_*k*_ to denote the index number of the *k*-th nonzero element of 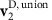, where 1≤*k*≤*m* and *m* denotes the number of nonzero elements. We then considered the projection matrix 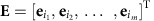 to the subspace, where 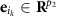 is the standard basis vector containing ‘1' in the *i*_*k*_-th element and ‘0' in the other elements. Finally, we derived the vector in the subspace **z**∈**R**^*m*^ by projecting the original connectivity vector **x**_2_ as


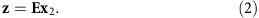


By choosing the FCs that corresponded to the canonical variables that are connected only with the diagnostic label, we could select essential FCs for classification. Simultaneously, undesirable effects caused by demographic and imaging differences at different imaging sites, that is NVs, were reduced through *L*_1_-SCCA, as explained in Supplementary Notes 6,4 and Supplementary Fig. 8. This procedure makes the MRI data from the three imaging sites useful in constructing a robust classifier that generalizes across ‘foreign', that is, USA, imaging sites.

### Prediction of the diagnostic label

To predict the diagnostic label from the extracted feature input **z** of equation (2) (identified FCs), we employed logistic regression as the classifier. In logistic regression, a logistic function is used to define the probability of a participant belonging to the ASD class as


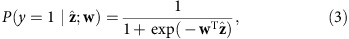


where *y* represents the diagnosis class label, that is, *y*=1 indicates ASD and *y*=0 indicates the TD class, respectively. 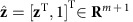 is a feature vector with an augmented input, where the feature vector **z** is extracted from the connectivity matrix of one participant's resting-state MRI sample (for more detail about data standardization, see Supplementary Note 7). Using the augmented input ‘1' is a standard approach to introduce constant (bias) input for the classifier. **w**∈**R**^*m*+1^ is the weight vector of the logistic function. To further decrease the dimension of the feature vector, which was already reduced by *L*_1_-SCCA according to equation (2), we used an SLR method, as described in the next paragraph. SLR automatically selects the features related to the ASD label as input for the logistic function. In SLR, the probability distribution of the parameter vector is estimated using the hierarchical Bayesian estimation approach, in which the prior distribution of each element of the parameter vector is represented as a Gaussian distribution. Because of the automatic relevance determination property of the hierarchical Bayesian estimation method, some of the Gaussian distributions become sharply peaked at zero so that the irrelevant features are not used in the classification.

### Linear regression to the clinical indices

Using the identified 16 FCs in the classifier, we predicted the four domain scores of the two standard diagnostic instruments measured: the ADOS^47^ and the ADI-R^48^ (see also Supplementary Table 3). Specifically, in each domain of an instrument, the score of each individual was predicted by calculating the linear weighted sum of his/her 16 correlation coefficients that corresponded to the 16 FCs in the classifier. The set of weights for this summation was determined through LOOCV, in which the domain scores of all-but-one participants were linearly regressed using the respective 16 correlation coefficients as explanatory variables. Because previous studies indicated age- and sex-related differences in cognition across the lifespan of adult ASDs^69^,^70^, behavioural measures such as ADOS and ADI-R can be assumed to exhibit dependence on these factors. We therefore incorporated age and sex into the regression model as additional explanatory variables. The LOOCV was necessary to avoid any information leakage from the individual to be predicted. The agreement between the measured and predicted domain scores was evaluated by the Pearson correlation coefficient and the statistical significance was tested against the null hypothesis that there is no relationship between measured and predicted scores. For the domain score with a significance correlation, the reliability of the prediction was further tested by a bootstrapping analysis (10,000 repetitions) where the alternative prediction was performed using 16 randomly selected FCs from the pool of 9,688 (=9,730−42) FCs, which were not selected by SLR in the LOOCV procedure for the ASD/TD classification (Supplementary Note 1). At each permutation, a new regression model was computed for each of the eight domain scores and the correlation between the predicted and measured domain score was calculated. The highest of the eight correlations (corresponding to the eight domains) was then selected and pooled over the permutations. The reliability of the prediction using the original 16 FCs was evaluated by integrating the normalized cumulative distribution of the pooled correlation coefficients derived through this bootstrapping procedure.

### Code availability

The classification code and the correlation matrix data used in the present study are available at a secure server of ATR Brain Information Communication Research Laboratory. Please contact the server administrator (asd-classifier@atr.jp) for access.

## Additional information

**How to cite this article:** Yahata, N. *et al*. A small number of abnormal brain connections predicts adult autism spectrum disorder. *Nat. Commun.* 7:11254 doi: 10.1038/ncomms11254 (2016).

## Supplementary Material

Supplementary Information Supplementary Figures 1-8, Supplementary Tables 1-9, Supplementary Notes 1-7 and Supplementary References

Supplementary Movie 1 Spatial distribution of the 16 FCs identified for the ASD/TD classifier as viewed from varying angle in a 3D space. See Fig. 2 and Table 1 for the definition of the node region numbers.

## Figures and Tables

**Table 1:** Properties of the 16 interregional FCs used in the classification of the ASD and TD populations.

**ID**	**Terminal regions**					**Mean FC**		**Wt.**
	**Lat.**	**Name**	**Gyral region**	**BA**	**Net.**	***r***_**TD**_	***r***_**ASD**_	
1	R	Diagonal ramus of the lateral f.	(4) Inferior frontal g.	44	SM	0.77	0.71	−0.88
	R	Ascending ramus of the lateral f.	(6) Inferior frontal g.	45	CO			
2	R	Subcallosal s.	(21) Middle cingulum	23	DM	0.39	0.22	−1.95
	R	Calloso-marginal posterior f.	(22) Middle cingulum	23	CO			
3	R	Thalamus	(28) Thalamus	—	CO	0.30	0.10	−2.62
	L	Subcallosal s.	(23) Posterior cingulum	29	DM			
4	R	Amygdala	(24) Amygdala	34	CO	0.16	0.05	−2.14
	L	Accumbens	(25) Caudate	—	CO			
5	R	Rhinal s.	(12) Parahippocampal g.	30	CO	0.11	−0.04	−2.11
	R	Olfactory s.	(8) Rectus	11	DM			
6	R	Median occipito-temporal lateral s.	(11) Inferior temporal g.	20	CO	0.03	−0.01	−0.98
	L	Anterior inferior frontal s.	(3) Inferior frontal g.	45	FP			
7	R	Posterior terminal ascending branch of the superior temporal s.	(10) Middle temporal g.	21	DM	−0.09	−0.21	−1.26
	R	Internal occipito-temporal lateral s.	(13) Fusiform	37	OC			
8	R	Intermediate precentral s.	(2) Middle frontal g.	46	FP	−0.10	−0.19	−1.59
	L	Lobe occipital	(16) Middle occipital g.	19	OC			
9	L	Polar frontal s.	(1) Superior frontal g.	9	DM	−0.16	−0.29	−1.52
	R	Retro central transverse ramus of the lateral f.	(15) Postcentral g.	3	SM			
10	R	Caudate	(26) Caudate	—	CO	0.17	0.22	0.76
	L	Calloso-marginal anterior f.	(20) Anterior cingulum	32	CO			
11	R	Olfactory s.	(8) Rectus	11	DM	−0.10	−0.02	1.78
	L	Parieto-occipital f.	(17) Cuneus	18	DM			
12	L	Pallidum	(27) Pallidum	—	CO	−0.14	0.04	1.90
	R	Superior temporal s.	(9) Superior temporal g.	22	CO			
13	M	Vermis	(29) Vermis	—	CB	−0.18	−0.04	1.85
	R	Superior temporal s.	(9) Superior temporal g.	22	CO			
14	L	Superior parietal s.	(14) Superior parietal g.	7	FP	−0.19	−0.06	0.99
	R	Anterior lateral f.	(7) Inferior frontal g.	47	CO			
15	R	Inferior precentral s.	(5) Inferior frontal g.	44	SM	−0.24	−0.13	1.00
	R	Parieto-occipital f.	(18) Cuneus	18	OC			
16	L	Anterior inferior frontal s.	(3) Inferior frontal g.	45	FP	−0.24	−0.16	1.74
	R	Calcarine f.	(19) Calcarine	17	OC			

CB, cerebellum; CO, cingulo-opercular; DM, default-mode; f., fissure; FC, functional connection; FP, fronto-parietal; g., gyrus; Lat, laterality; L, left; M, medial; Net, network; OC, occipital; R, right; s., sulcus; SM, sensorimotor; Wt., weight.

Listed here are the laterality and anatomical identification of the terminal regions (as defined in the Brainvisa Sulci Atlas), the associated gyral regions (as identified by the Anatomical Automatic Labeling), their Brodmann's areas, and associated networks, for each connection. The number in parenthesis appended to each gyral region represents the region identification in Fig. 2. Identification of the network is after Dosenbach *et al*.^14^. In addition, the mean correlations of the TD and ASD populations and the weighting coefficient in the SLR classifier are shown. FCs 1–9 represent under-connectivity (*r*_ASD_<*r*_TD_), whereas FCs 10–16 represent over-connectivity (*r*_ASD_>*r*_TD_). See also Supplementary Fig. 6.
