# Supplementary material for: A small number of abnormal brain connections predicts adult autism spectrum disorder
Source: Nat Commun. 2016 Apr 14;7:11254. doi: 10.1038/ncomms11254 (PMC4834637; doi:10.1038/ncomms11254)
Supplement: Supplementary Information — Supplementary Figures 1-8, Supplementary Tables 1-9, Supplementary Notes 1-7 and Supplementary References [file ncomms11254-s1.pdf]

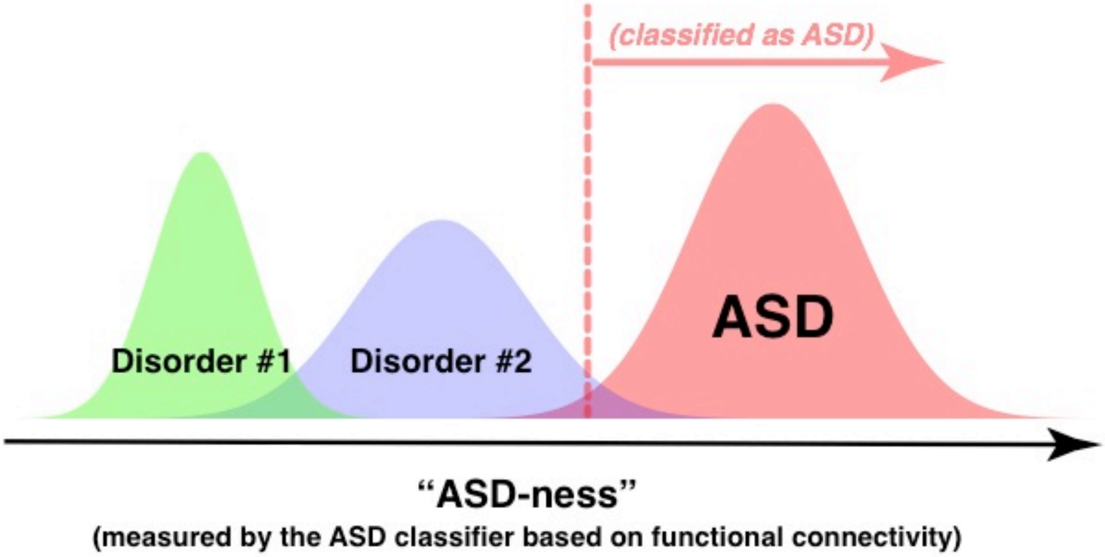

Supplementary Figure 1 | A schema illustrating the concept of "ASD-ness". It is proposed here that, at the individual level, the output of the ASD classifier with a good generalization across sites might provide a quantitative measure of "ASD-ness" along one of biological dimensions in psychiatric disorders. Applying such a measure to other disorders (such as schizophrenia, attention deficit hyperactivity disorder, major depressive disorder, etc.) may lead to a new possibility of quantifying spectral relationships among them, thereby bridging the categorical and biological dimension views of psychiatric disorders.

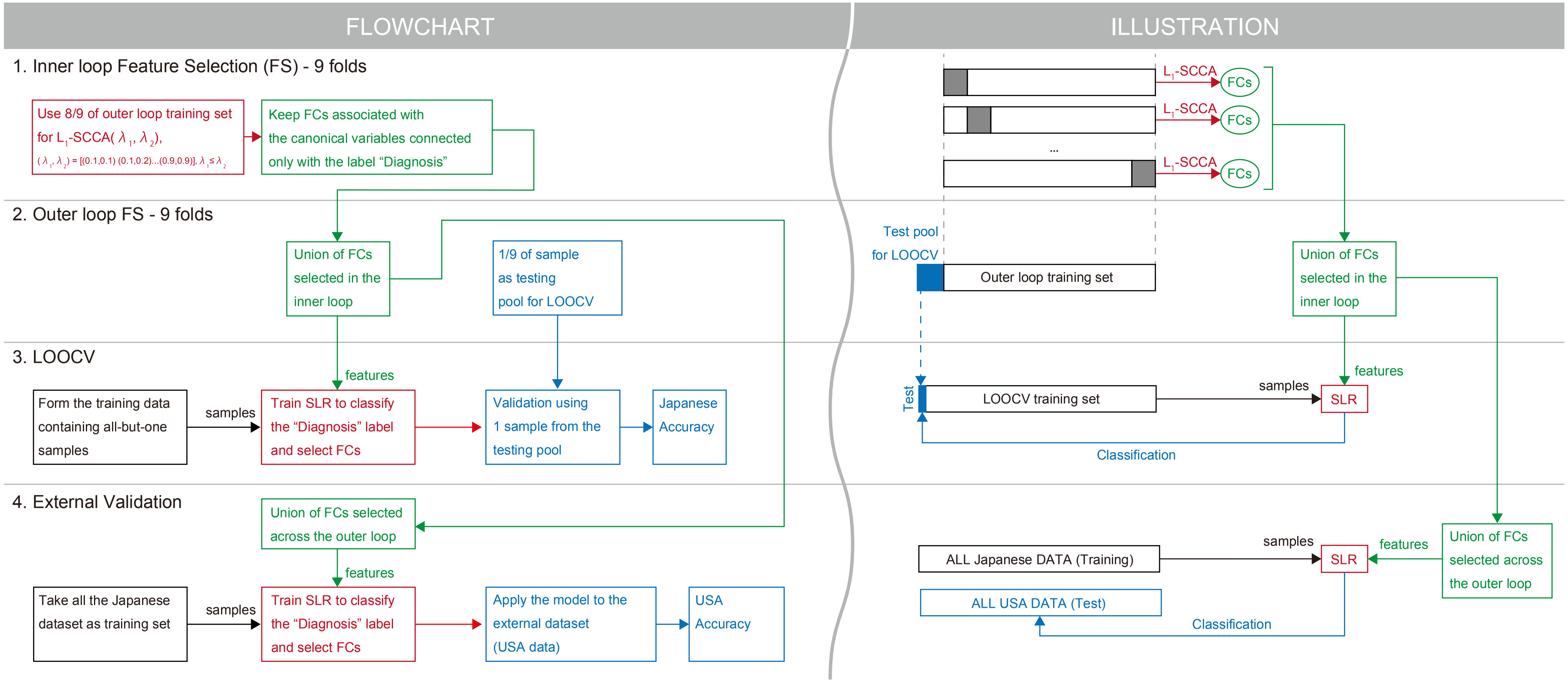

FLOWCHART
ILLUSTRATION
1. Inner loop Feature Selection (FS) - 9 folds
Use 8/9 of outer loop training set for L_1-SCCA(λ_1, λ_2), (λ_1, λ_2) = [(0.1,0.1) (0.1,0.2)...(0.9,0.9)], $\lambda_1 \leq \lambda_2$
Keep FCs associated with the canonical variables connected only with the label "Diagnosis"
2. Outer loop FS - 9 folds
Union of FCs selected in the inner loop
1/9 of sample as testing pool for LOOCV
3. LOOCV
Form the training data containing all-but-one samples
samples
features
Train SLR to classify the "Diagnosis" label and select FCs
Validation using 1 sample from the testing pool
Japanese Accuracy
4. External Validation
Union of FCs selected across the outer loop
features
Take all the Japanese dataset as training set
samples
Train SLR to classify the "Diagnosis" label and select FCs
Apply the model to the external dataset (USA data)
USA Accuracy
L_1-SCCA
FCs
...
Test pool for LOOCV
Outer loop training set
Union of FCs selected in the inner loop
Test
LOOCV training set
samples
features
SLR
Classification
ALL Japanese DATA (Training)
ALL USA DATA (Test)
samples
features
SLR
Union of FCs selected across the outer loop
Classification

Supplementary Figure 2 | Schematic diagram of the procedure for selecting FCs as ASD biomarker and assessing their predictive power. The left and right panels represent, respectively, the flowchart and illustration of the procedure. Black, blue, red, and green colors are conceptually associated with, respectively, training, testing, methods and features. (1) In each iteration of the inner loop feature selection (FS), 8/9 of the outer loop training set is used to train L_1-SCCA with different hyper-parameters. Functional connectivity features (FCs) that are associated with the canonical variables connected only with the label "Diagnosis" are retained. (2) In the outer loop FS, 1/9 of the samples is retained as testing pool for leave-one-out cross-validation (LOOCV), and the union of the FCs selected throughout the inner loop is derived. (3) One sample is taken from the testing pool of the outer loop, and used as test set of LOOCV. The remaining samples are used to train SLR on the union of the FCs retained during the inner loop. This procedure is repeated for every sample in the testing pool of the outer loop. In this way, the test set of LOOCV is always independent from the dataset used to select features. (4) The union of the FCs selected across the outer loop is used to train the final SLR on the whole Japanese dataset, and validated using an external cohort dataset (e.g. the US ABIDE dataset). In conclusion, nested feature selection is used to remove nuisance FCs, LOOCV is used to quantify generalizability on the Japanese dataset, and the external validation is used to quantify generalizability on the independent dataset.

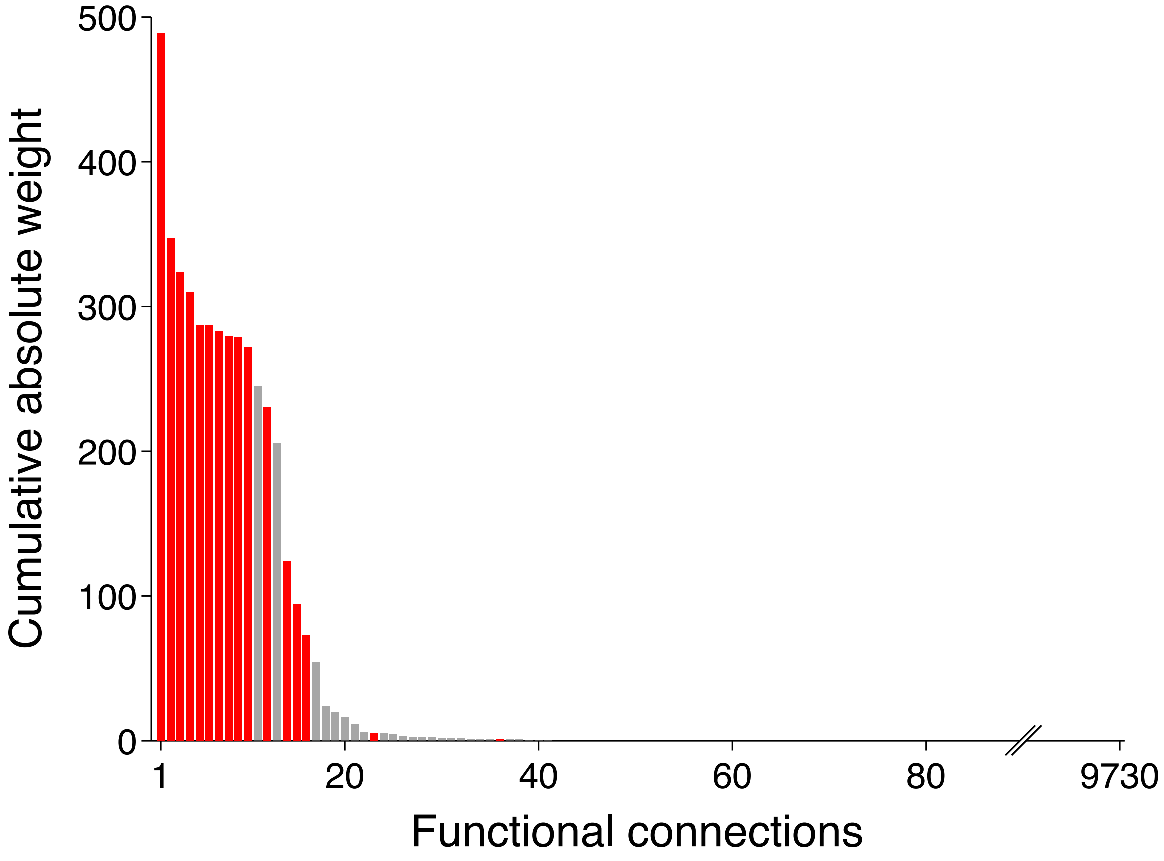

Supplementary Figure 3 | Contribution of each FC to the classification into ASD and TD. The cumulative absolute weights are shown for all of the 9,730 FCs. A greater magnitude of the cumulative absolute weight represents a larger degree of contribution by that FC to the classifiers. The 16 FCs identified by the final Japanese ASD/TD classifier constituted a very important subset of the 42 FCs that were selected at least once throughout the LOOCV (the 16 FCs are shown in red and the remaining 26 FCs are shown in gray). Thus, we confirmed the robustness and stability of the identified 16 FCs across 181 cross validation sets.

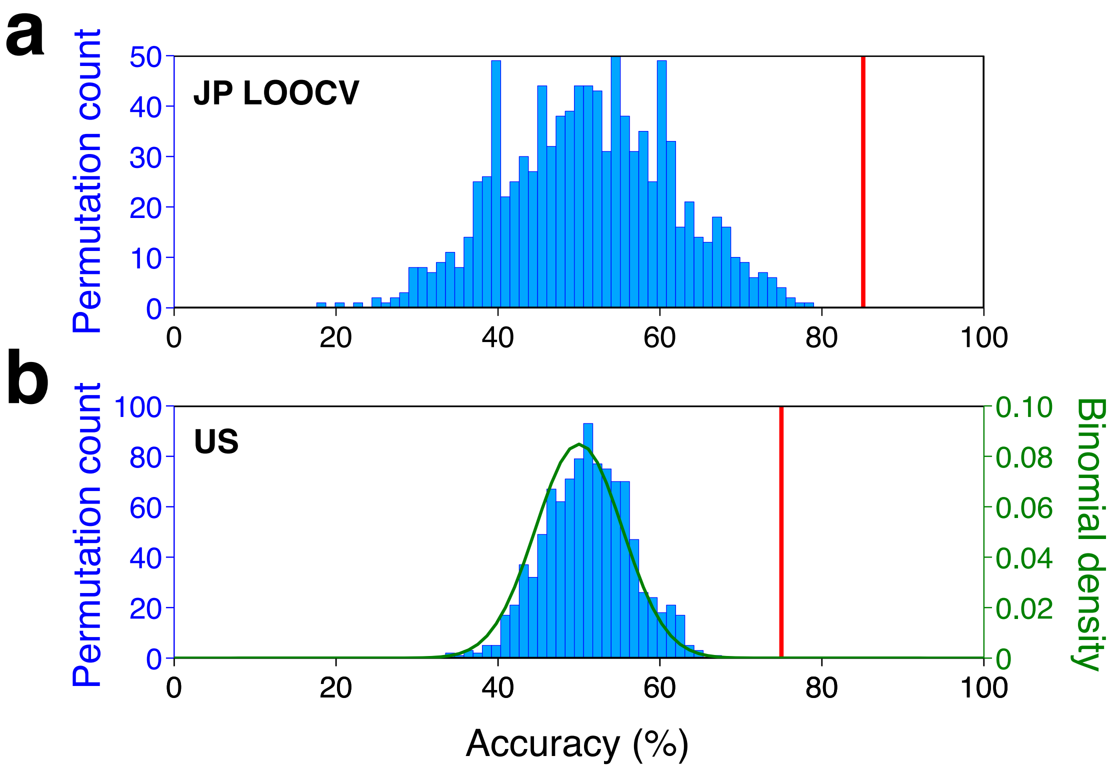

Supplementary Figure 4 | Permutation test. Panels **(a)** and **(b)** show the histograms of the permutation test (1,000 repetitions) for the JP LOOCV and the out-of-sample US accuracies, respectively. In panel **(b)**, the binomial distribution is shown as a green curve. The vertical red lines indicate the accuracy of the ASD classifier trained and tested without permutation. Both LOOCV and out-of-sample accuracies (i.e. US) were significant at $P = 0.001$, as demonstrated by the two panels. We observe that for the out-of-sample case [i.e. panel **(b)**] the binomial distribution is consistent with the permuted distribution. As suggested by Noirhomme *et al.* (2014)[1], the decreased independence among samples in LOOCV widens the permuted distribution relative to the binomial one; however, "with an independent validation set, the binomial test is perfectly valid[1]".

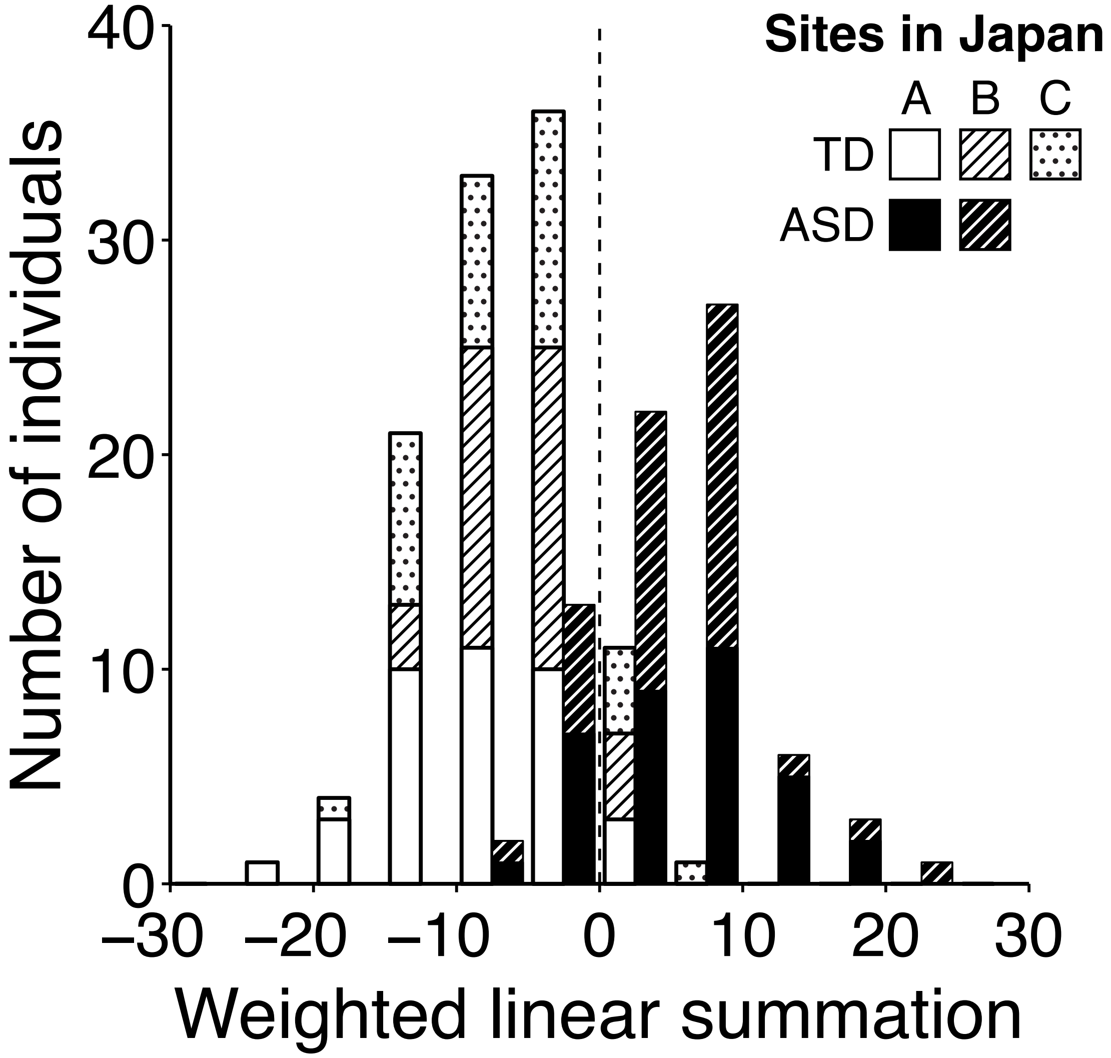

Supplementary Figure 5 | Numbers of TD (N=107) and ASD (N=74) individuals among all whose weighted linear summation (WLS) of the selected 16 FCs was within a specific WLS interval of width 5 for the three sites in Japan. The TD and ASD distributions of WLS are shown per site of data acquisition (A–C). The number of individuals is shown in the ordinate for a specific WLS interval of width 5. Those WLSs greater and less than zero are classified as ASD and TD, respectively. The inset shows symbols for the three imaging sites A, B and C and types of individuals (TD and ASD). Site A, University of Tokyo; site B, Showa University Karasuyama Hospital; site C, Advanced Telecommunications Research Institute International. The results indicate that the accuracy of the classification was equally high among the three sites (see also Supplementary Table 2).

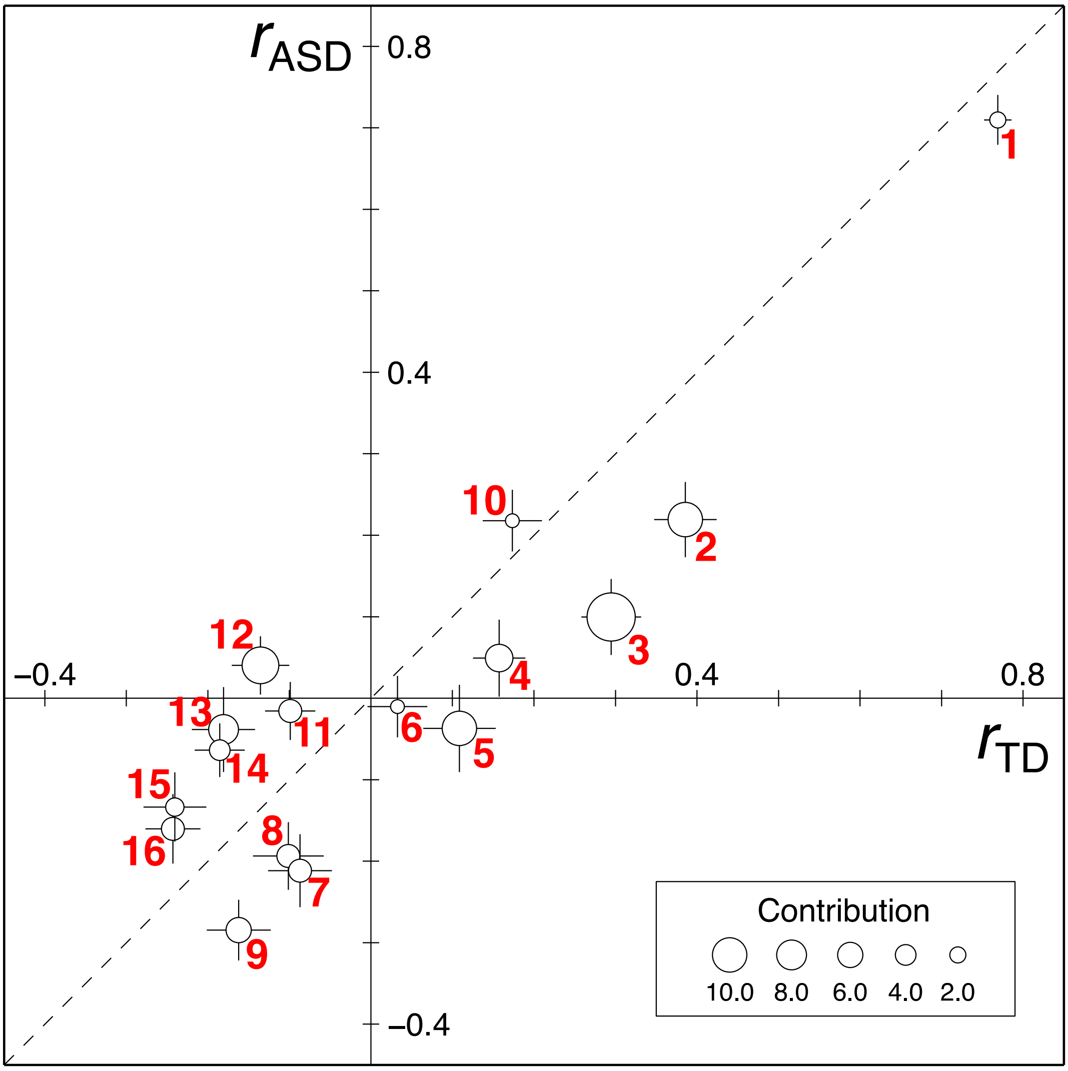

Supplementary Figure 6 | Plot of the mean interregional correlation values for the 16 FCs selected in the classifier for ASD individuals (ordinate) as a function of the corresponding correlation values for TD individuals (abscissa). The ASD and TD populations would possess equal mean correlation values for a FC on the diagonal dashed line. The 9 FCs below this line are FCs exhibiting under-connectivity ($r_{ASD} < r_{TD}$), whereas the 7 FCs above the line are FCs exhibiting over-connectivity ($r_{ASD} > r_{TD}$). An individual FC is represented by a circle, with the radius of the circle scaled by the contribution index of the corresponding connection as defined by the difference in the mean correlation values multiplied by the weight assigned in SLR (inset). The vertical and horizontal lines for each connection show the 95% confidence intervals of correlations for the ASD and TD groups, respectively. See Table 1 for the property of each FC.

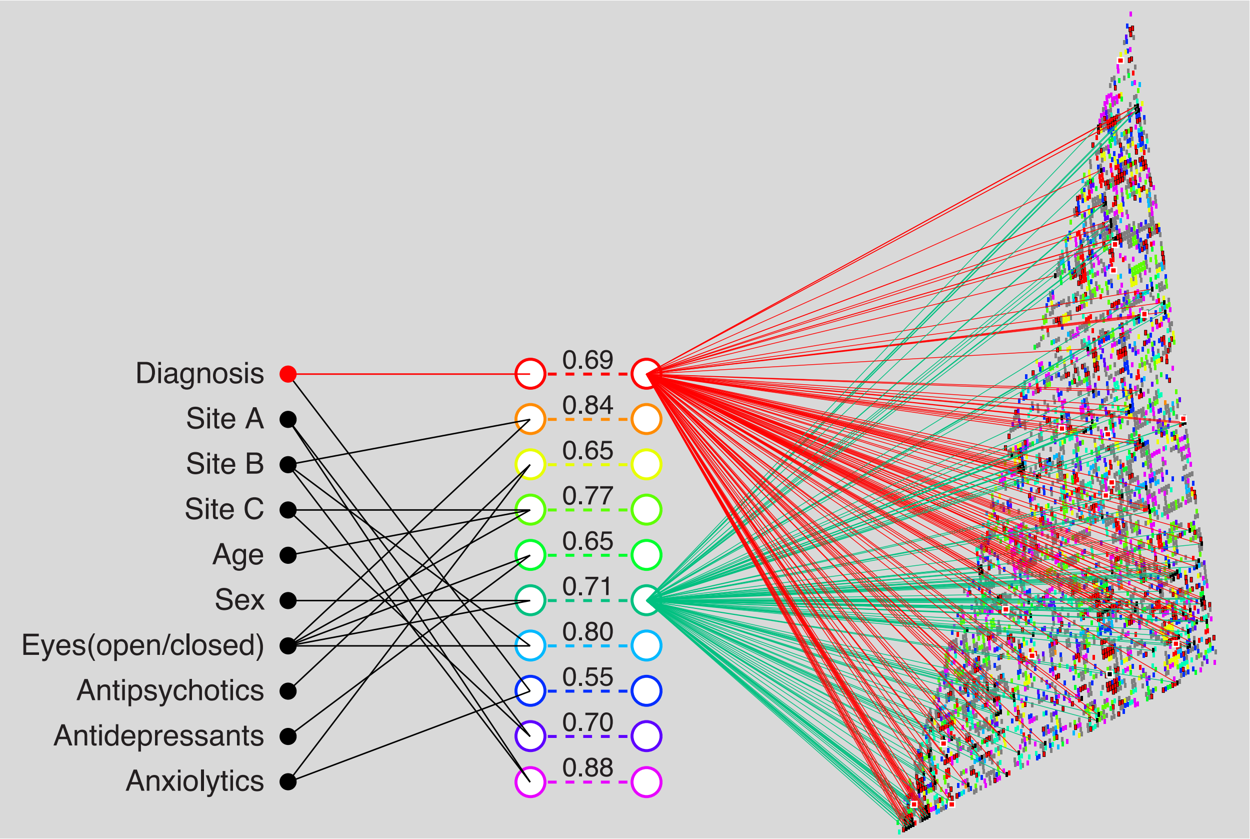

Supplementary Figure 7 | Extracted relationship between demographic information and functional connectivity using L_1-SCCA (next page). This figure represents one of the iterations of L_1-SCCA in the nested feature selection, where a canonical variable is connected only to "Diagnosis." As an example, here we show the smallest lambda combination (i.e. $\lambda_1 = 0.4$, $\lambda_2 = 0.2$) that yields at least one canonical correlation for each demographic information, in the first fold of the outer loop. The canonical variables are represented by white-open circles, encoded with different colors. The white-open circles in the left column indicate the canonical variable $\mathbf{v_1}^T\mathbf{x_1}$, derived from demographic information and imaging conditions; white-open circles in the right column indicate the canonical variable $\mathbf{v_2}^T\mathbf{x_2}$, which is derived from the functional connectivity (FC). The numbers on the dotted lines connecting canonical variables represent the correlation coefficients between $\mathbf{v_1}^T\mathbf{x_1}$ and $\mathbf{v_2}^T\mathbf{x_2}$. The connections between the demographic labels and canonical variables $\mathbf{v_1}^T\mathbf{x_1}$ are represented with black lines. If there is only one link towards a canonical variable, the color of the canonical variable is also used for the link (e.g. the link connecting "Diagnosis" and the 1st canonical variable is red). On the right of the figure, FCs are visualized and encoded with the color of the respective canonical variable. If canonical variables have overlapping FCs, those are colored in gray. However, if the overlap involves the 1st canonical variable (i.e. red) a red square with a black edge is used. In this example we focus on the overlapping between the 1st and the 6th canonical variables, representing "Diagnosis," and "Gender" and "Open/Closed Eye Condition", respectively. FCs that are common to these two canonical variables are represented with a black square and connected with a colored line to the respective canonical variable. The FCs identified by the SLR classifier on the whole Japanese dataset are represented with a white edge, filled with black if an overlap with the 6th canonical variable exists, and with red otherwise. The amount of FCs associated with the 1st canonical variable was 745 and the one associated with the 6th canonical variable was 659 with an overlap of 141 FCs. Moreover, the amount of FCs selected by SLR that overlapped with the 6th canonical variable was only 1. The lambda combination where a canonical variable has only one link to "Diagnosis" was on average 17.6±5.0% of the total amount of combinations. Moreover, we observe that lambda combinations larger than ($\lambda_1 = 0.4$, $\lambda_2 = 0.4$), never comply with this constraint. On average, the number of FCs associated with a "Diagnosis" canonical variable was 925±798.

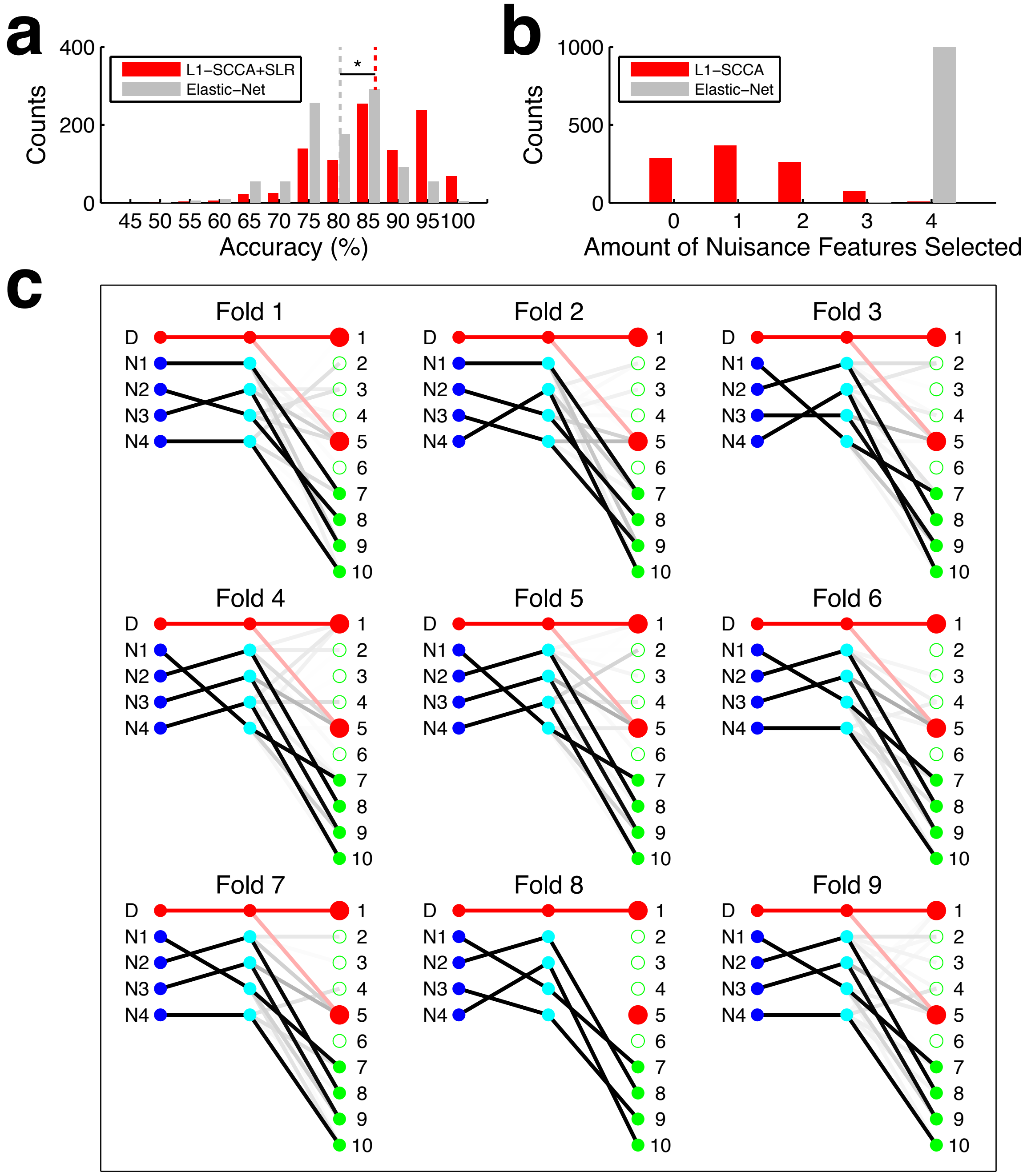

Supplementary Figure 8 | Simulation results using synthetic data (next page). This figure visualizes the results obtained from the analysis described in Supplementary Note 4. For the sake of readability, we discuss the synthetic dataset with the same terminology used for the real dataset. For example, "diagnostic label" means "synthetic diagnostic label". **(a)** Classification performance. Histograms depict the accuracy distribution, while the vertical dashed lines represent the mean accuracy of the two methods. Our proposed method, which uses L_1-SCCA for the feature selection, shows better classification performance (two-sample *t*-test, $P = 1.06\times10^{-52}$) than that of the standard elastic-net approach. **(b)** Amount of nuisance-related features (i.e. nuisance features) used to predict diagnostic label. The figure shows how frequently a given amount of nuisance features was selected by using the two different classification methods. The nuisance features were less frequently selected by using our proposed method than by using elastic-net. These results indicate effectiveness of L_1-SCCA for eliminating nuisance features. **(c)** Instance of the L_1-SCCA procedure. Each subpanel represents the transformation matrices obtained by L_1-SCCA in a given nested fold. Here we

visualize the $< \lambda_1, \lambda_2 >$ combination where the *diagnostic canonical constraint* was last met (for more details see Supplementary Note 4). The red color is used when an entity (i.e. line or dot) is related to the diagnostic label (*D)*. The nuisance variables are represented by the blue dots labeled as *N1, N2, N3, N4,* and together with the diagnostic label (*D)* they form the demographic information. The elements of connectivity input are represented by the nodes labeled from 1 to 10. The canonical variables are represented by the cyan dots between the demographic information and the features. Filled green dots represent the nuisance features. White-open green circles depict the features with zero contribution to any demographic information. The color intensity of the lines is proportional to the connection strength (i.e. absolute value of the weight). We observe that the *diagnostic canonical constraint* of having one canonical variable assigned exclusively to the diagnostic label is met. Moreover, the canonical variables assigned to the nuisance variables always have the strongest connection with the nuisance features (i.e. 7–10). Fold 8 shows a missing connection between one of the clean features (i.e. 5) and the canonical variable assigned to the diagnostic label. However, the missing feature in Fold 8 is selected by other folds, highlighting the usefulness of the nested subsampling procedure.

Supplementary Table 1 | Demographic information of the participants used to construct the rs-fcMRI-based classifier of the ASD and TD populations (mean ± SD). All demographic distributions between ASD and TD populations in the Japanese and USA data are matched ($P > 0.05$).

	Site A		Site B		Site C
	ASD	TD	ASD	TD	TD
Male/Female	23/12	20/18	35/4	30/6	23/10
Age (yr)	31.9 ± 8.8	35.4 ± 7.4	31.0 ± 8.2	30.9 ± 6.9	24.2 ± 5.3
Handedness	91.9 ± 13.2	92.8 ± 15.3	87.9 ± 27.7	95.1 ± 18.3	right-handed
IQ	107.1 ± 13.2	106.6 ± 8.1	110.2 ± 8.5	109.2 ± 8.4	NR

NR, not recorded.

Supplementary Table 2 | Summary of the classification performance evaluated at each of three imaging sites (A–C) in Japan.

	Site A	Site B	Site C	Total
Subjects (ASD/TD)	35/38	39/36	0/33	74/107
Accuracy (%)	85	85	85	85
Sensitivity (%)	77	82	–	80
Specificity (%)	92	89	85	89
Diagnostic odds ratio (DOR)	39.4	36.5	–	31.1

Note that in site C, individuals with ASD were not recruited, thus sensitivity and DOR cannot be evaluated.

Supplementary Table 3 | Prediction of the measured domains of the two diagnostic instruments, Autism Diagnostic Observation Schedule (ADOS) and Autism Diagnostic Interview-Revised (ADI-R). In each domain, the score of each individual was predicted by computing a linear weighted summation of a subset within the 16 FCs included in the classifier. The Pearson correlation coefficients (*r*) between the measured and predicted scores are shown. The statistical significance (*P*) is indicated both as uncorrected and as Bonferroni-corrected for multiple comparisons among the 8 domains.

Instrument	Domain	Content	*r*	*P*	
				Uncorrected	Corrected
ADOS ($N = 58$)	A	Communication	0.442	0.001	0.008
	B	Reciprocal social interaction	0.159	0.234	(1)
	C	Imagination and creativity	0.146	0.274	(1)
	D	Stereotyped behaviors and restricted interests	0.062	0.644	(1)
ADI-R ($N = 27$)	A	Reciprocal social interaction	0.453	0.018	0.144
	B	Abnormalities in communication	0.389	0.045	0.360
	C	Restricted, repetitive, and stereotyped patterns of behavior	0.053	0.793	(1)
	D	Abnormality of development evident at or before 36 months	–0.013	0.948	(1)

Supplementary Table 4 | Classification performances for the Japanese discovery cohort and the USA independent validation cohort when only a subset of the Japanese three sites was used for training the ASD classifier.

Dataset	Accuracy (%)					
	Site 1	Site 2	Site 1+Site 2	Site 1+Site 3	Site 2+Site 3	Mean ± SD
JP LOOCV	75.3	48.0	83.8	68.9	67.6	68.7 ± 13.2
US Generalization	53.4	52.3	65.7	63.6	73.9	61.8 ± 9.0

Supplementary Table 5 | Summary of imaging protocols for resting-state fMRI at the imaging sites listed in Supplementary Table 1.

Parameter	Site			
	A (TD)	A (ASD)	B	C
MRI scanner	Philips Achieva	Philips Achieva	GE Signa	Siemens MagnetomTrio
Magnetic field strength (T)	3.0	3.0	1.5	3.0
Field of view (mm)	224	220	220	192
Matrix	64 × 64	80 × 80	64 × 64	64 × 64
Number of slices	45	34	27	33
Number of volumes	200	200	204	150
In-plane resolution (mm)	3.5 × 3.5	2.75 × 2.75	3.4375 × 3.4375	3.0 × 3.0
Slice thickness (mm)	3.5	5.0	5.0	3.5
Slice gap (mm)	0.0	0.0	1.0	0.0
TR (ms)	2,500	2,500	2,000	2,000
TE (ms)	30	30	30	30
Total scan time (mm:ss)	8:20	8:20	6:48	5:00
Flip angle (deg)	75	75	90	80
Slice acquisition order	Ascending	Ascending	Ascending (interleaved)	Ascending (interleaved)
Instructions to participants and other imaging conditions	'Please relax during the scan. Do not think of anything in particular, do not sleep, but keep looking at the crosshair mark presented'. The lights in the scan room were dimmed.	'Please relax during the scan. Do not sleep'. Instructions regarding the eyes were either 'you may close your eyes if you want' (*N*=23) or 'please close eyes during the scan' (*N*=12). The scan was conducted in a dark room.	'During the scan, please close your eyes, do not think of anything in particular, and stay still. Please do not sleep'. The lights in the scan room were dimmed.	'Please relax during the scan. Do not sleep and keep looking at the fixation point presented (a tiny double circle). Do not think of anything in particular'. The lights in the scan room were dimmed.

Supplementary Table 6 | Detailed characteristics of the head motion of the ASD and the TD populations in the Japanese and the USA data.

		Japanese (training data)			US ABIDE (test data)		
		ASD	TD	*P*	ASD	TD	*P*
Translation[a]	*x*	0.013 ± 0.006	0.015 ± 0.012	0.861	0.022 ± 0.013	0.019 ± 0.009	0.370
	y	0.043 ± 0.036	0.038 ± 0.030	0.980	0.056 ± 0.034	0.044 ± 0.022	0.128
	z	0.040 ± 0.030	0.047 ± 0.033	0.216	0.069 ± 0.050	0.061 ± 0.035	0.773
Rotation[a,b]	*x*	0.027 ± 0.013	0.033 ± 0.022	0.260	0.046 ± 0.039	0.036 ± 0.019	0.825
	y	0.012 ± 0.006	0.015 ± 0.014	0.470	0.018 ± 0.013	0.014 ± 0.006	0.299
	z	0.011 ± 0.005	0.010 ± 0.005	0.201	0.017 ± 0.010	0.013 ± 0.005	0.192

([a]mean relative displacement in units of mm; [b]head radius is assumed to be 50 mm.)

Supplementary Table 7 | Demographic information of the participants selected from the USA ABIDE Project (mean ± SD).

Site ID	ASD			TD		
	Age (yr)	Sex (M/F)	FIQ	Age (yr)	Sex (M/F)	FIQ
CAL	23.9 ± 6.2	5/2	103.0 ± 10.6	26.5 ± 8.3	4/2	114.7 ± 10.8
CMA	21.5 ± 0.7	2/0	117.5 ± 13.4	22.3 ± 2.3	3/0	106.7 ± 4.9
NYU	19.6	1/0	94.0	19.1	1/0	107.0
OLN	19.0 ± 1.4	2/0	109.0 ± 12.7	20.5 ± 0.7	2/0	116.5 ± 20.5
PIT	23.2 ± 4.4	6/0	118.2 ± 16.5	22.6 ± 3.3	6/0	113.5 ± 11.4
TTY	20.8 ± 2.6	8/0	114.5 ± 11.2	21.2 ± 2.6	8/0	115.1 ± 10.2
USM	26.9 ± 8.3	18/0	108.1 ± 14.0	25.8 ± 4.9	18/0	113.2 ± 14.3
TOTAL	24.0 ± 6.6	42/2	110.0 ± 13.6	24.0 ± 5.0	42/2	113.3 ± 12.0

CAL, California Institute of Technology; CMA, Carnegie Mellon University; NYU, New York University; OLD, Olin, Institute of Living at Hartford Hospital; PIT, University of Pittsburgh School of Medicine; TTY, Trinity Centre for Health Sciences; USM, University of Utah School of Medicine.

Supplementary Table 8 | Imaging protocols for resting-state fMRI used at the imaging sites listed in Supplementary Table 7.

Parameter	Site						
	CAL	CMA	NYU	OLN	PIT	TTY	USM
MRI scanner	Siemens Magnetom Trio	Siemens Magnetom Verio	Siemens Magnetom Allegra	Siemens Magnetom Allegra	Siemens Magnetom Allegra	Philips Achieva	Siemens Magnetom Trio
Magnetic field strength (T)	3.0	3.0	3.0	3.0	3.0	3.0	3.0
Field of view (mm)	224	192	240	220	200	240	220
Matrix	64 × 64	64 × 64	80 × 80	64 × 64	64 × 64	80 × 80	64 × 64
Number of slices	34	28	33	29	29	38	40
Number of volumes	150	240	180	210	200	150	240
In-plane resolution (mm)	3.5 × 3.5	3.0 × 3.0	3.0 × 3.0	3.4375×3.4375	3.125 × 3.125	3.0 × 3.0	3.4375×3.4375
Slice thickness (mm)	3.5	3.0	4.0	4.0	4.0	3.5	3.0
Slice gap (mm)	0.0	1.5	0.0	1.0	0.0	0.35	0.3
TR (ms)	2000	2000	2000	1500	1500	2000	2000
TE (ms)	30	30	15	27	25	28	28
Total scan time (mm:ss)	5:04	8:06	6:00	5:15	5:06	5:06	8:06
Flip angle (deg)	75	73	90	60	70	90	90
Slice acquisition order	Ascending (interleaved)	Ascending (interleaved)	Ascending (interleaved)	Ascending (interleaved)	Ascending (interleaved)	Ascending	Ascending (interleaved)
Eyes during scan	Closed	Closed	Open	Open	Closed	Closed	Open

CAL, California Institute of Technology; CMA, Carnegie Mellon University; OLD, Olin, Institute of Living at Hartford Hospital; PIT, University of Pittsburgh School of Medicine; TTY, Trinity Centre for Health Sciences; USM, University of Utah School of Medicine.

Supplementary Table 9 | Summary of imaging protocols for resting-state fMRI in extra datasets of (a) ASD, (b) schizophrenia, (c) major depressive disorder, and (d) attention deficit hyperactivity disorder.

(a) ASD

Parameter	Site B*
MRI scanner	Siemens
Magnetic field strength (T)	3.0
Field of view (mm)	212
Matrix	64 × 64
Number of slices	40
Number of volumes	240
In-plane resolution (mm)	3.3 × 3.3
Slice thickness (mm)	3.2
Slice gap (mm)	0.8
TR (ms)	2,500
TE (ms)	30
Total scan time (mm:ss)	10:00
Flip angle (deg)	80
Slice acquisition order	Ascending
Instructions to participants and other imaging conditions	'Please relax. Do not think of anything in particular, do not sleep, but keep looking at the crosshair mark presented'. The lights in the scan room were dimmed.

(*Showa University Karasuyama Hospital, Japan)

(b) Schizophrenia

Parameter	MR Scanner*	
	#1	#2
MRI scanner	Siemens Trio	Siemens TimTrio
Magnetic field strength (T)	3.0	3.0
Field of view (mm)	256	212
Matrix	64 × 48	64 × 64
Number of slices	30	40
Number of volumes	180	240
In-plane resolution (mm)	4.0 × 4.0	3.3125 × 3.3125
Slice thickness (mm)	4.0	3.2
Slice gap (mm)	0	0.8
TR (ms)	2,000	2,500
TE (ms)	30	30
Total scan time (mm:ss)	6:00	10:00
Flip angle (deg)	90	90
Slice acquisition order	Ascending	Ascending (Interleaved)
Instructions to participants and other imaging conditions	Please relax. Fixate on the central crosshair mark and do not think of anything in the resting. The lights in the scan room were dimmed.	Same as Scanner #1.

(*Kyoto University Hospital, Japan)

Supplementary Table 9 (continued)

(c) Major depressive disorder

Parameter	Site*			
	#1	#2	#3	#4
Participants (MDD/HC)	57 / 66	8 / 47	23 / 29	17 / 3
MRI scanner	GE Signa HDxt	GE Signa HDxt	Siemens Magnetom	Siemens Verio
Magnetic field (T)	3.0	3.0	3.0	3.0
Field of view (mm)	256	256	192	212
Matrix	64 × 64	64 × 64	64 × 64	64 × 64
Number of slices	32	32	38	40
Number of volumes	150	150	112	244
In-plane resolution (mm)	4.0 × 4.0	4.0 × 4.0	3.0 × 3.0	3.3125 × 3.3125
Slice thickness (mm)	4.0	4.0	3.0	3.2
Slice gap (mm)	0	0	0	0.8
TR (ms)	2,000	2,000	2,700	2,500
TE (ms)	27	27	31	30
Total scan time (mm:ss)	5:00	5:00	5:03	10:10
Flip angle (deg)	90	90	90	80
Slice acquisition order	Ascending (Interleaved)	Ascending (Interleaved)	Ascending (Interleaved)	Ascending
Instructions to participants and other imaging conditions	Please relax. Do not think of anything in particular, do not sleep, but keep looking at the crosshair mark presented. The lights in the scan room were dimmed.	Same as Site 1	Same as Site 1	Same as Site 1

(*Site #1, the Hiroshima University Hospital, Japan; #2, the Hiroshima City General Rehabilitation Center, Japan; #3, the Kajikawa hospital, Japan; #4, the Hiroshima University KANSEI Innovation Center, Japan)

(d) Attention deficit hyperactivity disorder

Parameter	NeuroImage (ADHD-200)*
MRI scanner	Siemens Magnetom
Magnetic field strength (T)	3.0
Field of view (mm)	224
Matrix	64 × 64
Number of slices	37
Number of volumes	261
In-plane resolution (mm)	3.5 × 3.5
Slice thickness (mm)	3.0
Slice gap (mm)	0.5
TR (ms)	1,960
TE (ms)	40
Total scan time (mm:ss)	8:32
Flip angle (deg)	80
Slice acquisition order	Ascending (Interleaved)
Instructions to participants and other imaging conditions	Participants were asked to think of nothing in particular while keeping their eyes closed. No visual stimulus was presented during the scan.

(*see http://fcon_1000.projects.nitrc.org/indi/adhd200/)

Supplementary Notes

Supplementary Note 1

Importance of the final 16 FCs throughout all FCs selected in the LOOCV.

The 16 FCs incorporated in our final classifier were selected by SLR using the whole Japanese dataset, starting from a subset of FCs that was previously reduced by nested feature selection. One might wonder whether the 16 FCs that were finally identified were also frequently selected, with a large weight, throughout the LOOCV procedure. This is an important question regarding the stability and robustness of the finally identified 16 FCs. To answer this question, we define the cumulative absolute weight for the k-th FC (k = 1, 2, ..., 9730) in the form

$$c^k = \sum_{i=1}^{N} \left| w_i^k \right|,$$

where N=181 is the number of LOOCV folds (i.e. the number of subjects), and w_i^k is the weight associated with the k-th FC during the i-th LOOCV fold.

The greater magnitude of c^k indicates a more significant contribution by the k-th FC to the classification into ASD and TD, throughout the LOOCV. Supplementary Fig. 3 shows the magnitude distribution of the 42 nonzero instances of c^k. Sorting by their magnitudes, we found that the identified 16 FCs represent an important subset of the 42 FCs that were selected at least once during the LOOCV. Consequently, we conclude that the finally identified 16 FCs were stable and robust with respect to 181 LOOCV subsets of individuals and can be regarded as trustworthy.

Supplementary Note 2

Application of the ASD classifier to the extended ABIDE dataset including individuals with diverse profiles.

The goal of the present study was to establish a generalizable rs-fcMRI-based classifier by evaluating its performance using independent populations with well-defined profiles. An additional interest arises as to how the classifier works on individuals with other varying confounding factors such as presence of medication and comorbidity. To address this, we performed a supplementary analysis by applying the ASD classifier to the "extended" ABIDE dataset that incorporated individuals with diverse profiles. This dataset was formed by relaxing the selection criteria we adopted in our main analysis (see "Generalization to USA data" in Methods). Specifically, removing the conditions for the FIQ, comorbidity, and medication status, we additionally identified 19 individuals with ASD and demographically matched 19 TDs in the ABIDE data pool. We appended these individuals to the main dataset to form the extended ABIDE dataset that consisted in a total of 63 individuals with ASDs and 63 TDs. Repeating the same analysis for this extended dataset, we found: AUC = 0.74,

accuracy = 71%, sensitivity = 75%, specificity = 68%, and DOR = 6.4. Importantly, there was no statistically significant difference in the classifier's sensitivity between the original ASD population (N = 44, 75%) and the appended ASD population (N = 19, 74%) (chi-square test, P = 0.91). To that extent, this supplementary analysis indicates, therefore, that the influence of such confounding factors as FIQ, comorbidity, and medication status on the classification performance appeared to be minimal.

Supplementary Note 3

Comparison with the elastic net.

Our ASD/TD classifier attained accuracies of 85% for the Japanese dataset and 75% for the USA dataset. For purposes of comparison, we also applied to our data sets a state-of-the-art regularized (logistic) regression method called elastic net[2], which was utilized in a previous study[3].

Logistic regression was regularized by the elastic net, implemented by the *lassoglm* function in MATLAB. The inner loop of a 9×9 nested cross-validation was used to select the hyperparameters of the elastic net. Specifically, the α parameter was varied between 0.1, 0.5, 0.7, 0.9, 0.95, 0.99 and 1, in order to have more values close to 1 (i.e., toward Lasso). For the λ parameter (i.e. larger λ cause more sparsity), the largest value of λ that gives a non-null model was automatically found by *lassoglm.* The smallest λ was set to 10^{-2} of the largest λ. Subsequently, a sequence of 25 λ's between the smallest and the largest was used as the elastic net λ parameter. In the LOOCV, the medians of the α and λ parameters that yielded the highest AUC across the internal fold were used as elastic net hyperparameters. For the external validation utilizing the USA data set, the medians of the α and λ parameters summarized in the external fold (i.e. the median of the median) were used. The results of the LOOCV were AUC = 0.89 and Accuracy = 80%. The generalization to the external USA dataset was AUC = 0.73 and Accuracy = 61% with 173 finally selected FCs. The performance for the Japanese discovery cohort was comparable to our classifier but the accuracy for the USA independent validation cohort was 14% worse than our classifier, thereby showing much less generalization capability. These results clearly show the usefulness of our feature extraction and classification approach in preventing interferential effects by NVs because the elastic net algorithm did not explicitly avoid features related to NVs.

Supplementary Note 4

Effectiveness of L_1-SCCA in avoiding nuisance variables.

Eliminating the unwanted effects of nuisance variables on FCs is indispensable for a study using multi-center imaging data. This is because, in the absence of a "gold standard" method for rs-fcMRI

data acquisition, different sites adopt different scanning protocols and imaging instruments, which may exert significant effects on the measurement of FCs. In addition, the training of a reliable classifier requires a dataset with a large number of subjects at each site, which makes it difficult to equate all the demographic variables including diagnostic label, age, sex, etc., among multiple sites. Under such conditions, the diagnostic label and other nuisance variables may be correlated. Therefore, in order to achieve high generalization ability across multiple sites, it is essential to explicitly eliminate the unwanted effects of nuisance variables.

To illustrate this issue, we conducted a simplified simulation using synthetic data and visualized how L_1-SCCA performed. For the sake of readability, we discuss the synthetic dataset with the same terminology used for the real dataset. For example, “diagnostic label” means “synthetic diagnostic label”. Moreover, to keep consistency with the methods section, we define the matrix containing demographic variables as $\mathbf{X}_1$ and the matrix containing the connectivity input as $\mathbf{X}_2$.

We consider a 10-dimensional connectivity input to depict how L_1-SCCA performs with 100 samples (i.e. $\mathbf{X}_2 \in \mathbf{R}^{100\times 10}$). Each sample was independently generated from an identical Gaussian distribution with zero mean and unit covariance. Then, we divide the 100 samples into 70 training data samples and 30 test data samples. Here, we assume that two elements of the 10-dimensional input are related to diagnostic label, and other four elements are related to the nuisance variables. We used a weight vector of the form:

$$\mathbf{w}=\left[\mathrm{w}_1,0,0,0,\mathrm{w}_5,0,\mathrm{w}_7,\mathrm{w}_8,\mathrm{w}_9,\mathrm{w}_{10}\right]^{\mathrm{T}}$$

to synthesize the diagnostic label as $\mathbf{y} = \mathrm{sign}(\mathbf{X}_2\mathbf{w})$. In the weight vector $\mathbf{w}$, w_1 and w_5 correspond to the contribution of the two elements truly related to the diagnostic label (i.e. *clean*), and $\mathrm{w}_{7,8,9,10}$ correspond to the contribution of the four nuisance-related features (i.e. *nuisance*). When defining the elements of $\mathbf{w}$, we refer to the percentage of contribution with respect to the sum of the weights $\sum_{i=1}^{10} w_i$. We prepared two different weight vectors for the training and test data respectively. For the weight vector used to generate the training set ($\mathbf{w}_{\mathrm{tr}}$), $\mathrm{w}_{1,5,7,8,9,10}$ were sampled from a standard uniform distribution. Considering that the margin d between *clean* and *nuisance* weights is defined as

$$d = \min_{i=1,5} \mathrm{w}_i - \max_{i=7...10} \mathrm{w}_i,$$

we adopted the sampled weight vector if the margin d is larger than 10% and smaller than 20%. The lower bound constraint is necessary to keep the problem of training feasible, while the upper bound is required for keeping it non-trivial. On the other hand, the weights used to generate the test data

were $\mathbf{w}_{\mathrm{te}}$, where $\mathrm{w}_{1,5} = 50\%$ and $\mathrm{w}_{7,8,9,10} = 0$. Since $\mathbf{w}_{\mathrm{tr}}$ is designed to include elements related to the four nuisance variables, the corresponding diagnostic labels result in partially depending on these nuisance variables. For example, ASD occurs more frequently for males than females. In other words, the synthetic training set includes artifactual interference. On the other hand, since $\mathbf{w}_{\mathrm{te}}$ does not include nuisance elements, in the test dataset the diagnostic labels do not depend on the nuisance variables. Therefore, we can consider the test data as a clean dataset. With the synthesized data, we can evaluate how classification algorithms robustly predict the diagnostic label.

For the L_1-SCCA algorithm, we consider a 5-dimensional demographic target that includes diagnostic label and the four nuisance variables $\mathbf{X}_1 = \left[\mathbf{y},\ \mathbf{x}_1^2, \mathbf{x}_1^3, \mathbf{x}_1^4, \mathbf{x}_1^5\right]$. Supposing that also nuisance variables are generated with a vector of the form $\mathbf{w}$, the first nuisance variable is continuous and it is obtained by the following equation $\mathbf{x}_1^2 = \mathbf{X}_2\mathbf{w}$, where $\mathrm{w}_7 = 100\%$ and $\mathrm{w}_{i \neq 7} = 0$. The second nuisance variable is discrete and it was generated as $\mathbf{x}_1^3 = \mathrm{sign}(\mathbf{X}_2\mathbf{w})$, where $\mathrm{w}_k = 100\%$ and $\mathrm{w}_{i \neq k} = 0$ with $k = 8$. The same was done for $\mathbf{x}_1^4$ *and* $\mathbf{x}_1^5$ with $k = 9$ and $k = 10$, respectively. These four variables could correspond to age (continuous variable), sex (binary variable) and two site labels (binary variables). The simulation consisted of 1,000 repetitions. At each repetition, we applied the proposed method and elastic net to newly resampled $\mathbf{X}_2$ and $\mathbf{w}_{\mathrm{tr}}$.

As a result, we found that the classification performance of our proposed method which uses L_1-SCCA for the feature selection was better (two-sample t-test P = 1.06×10^{-52}) than that of the standard elastic-net approach (see Supplementary Fig. 8A). We also compared how frequently the nuisance-related features were selected by the two algorithms for predicting diagnostic labels (see Supplementary Fig. 8B). We then found that the nuisance-related features were less frequently selected by using our proposed method than by using elastic-net. This result showed the effectiveness of the L_1-SCCA in avoiding the influence of nuisance variables.

To concretely show how L_1-SCCA performed, we visualized the transformation matrix from demographic to canonical variables, and from connectivity inputs to canonical variables as a graph, for every nested fold in one repetition of the simulation (see Supplementary Fig. 8C). At each nested fold, we considered the $\langle \lambda_1, \lambda_2 \rangle$ combination where the *diagnostic canonical constraint* was last met (i.e. last iteration across $\langle \lambda_1, \lambda_2 \rangle$). The *diagnostic canonical constraint,* used in the feature selection procedure*,* determines that at least one canonical variable is assigned only to the diagnostic label (for details see the subsection of Methods entitled "L_1-regularized sparse canonical correlation analysis used in inner loop feature selection").

We observe from Supplementary Fig. 8C that the *diagnostic canonical constraint* was met.

Moreover, we found that the canonical variables assigned to the nuisance variables always have the strongest connection with the nuisance-related features. We also verified the usefulness of the nested subsampling procedure, where the union of the features selected across nested folds is considered, in order to obtain a stable and clean set of features. Specifically, Fold 8 in Supplementary Fig. 8C shows a missing connection between one of the clean features and the canonical variable assigned to the diagnostic label. In this way, if features were selected only based on Fold 8, the algorithm would have missed one feature, leading to a bad prediction. However, the union of features across folds is able to overcome the issue.

Supplementary Note 5

Generalization performance of the ASD classifier from the USA dataset to the Japanese dataset.

We trained the classifier using the USA dataset and then tested on the Japanese dataset. The results showed poorer classification performance (US LOOCV: 48%, Generalization to JP: 62%), and all the selected FCs were different from the 16 FCs that were extracted in our study (see also the Results section "Characteristics of the 16 identified FCs incorporated in the classifier"). This result is somewhat consistent with the classification performances described in Supplementary Table 4. Indeed, the total number of samples and the number of samples per site seem to play a crucial role in deriving a biomarker with high accuracy. With this in mind, we observe that the US ABIDE dataset has a total number of samples which is half of the Japanese dataset. Moreover, the number of samples per site is limited and highly variable in the ABIDE dataset compared to the Japanese dataset, on average: 12.6 ± 11.6 (USA dataset) vs. 60.3 ± 23.7 (Japanese dataset).

Supplementary Note 6

Relationship between demographic information and functional connectivity.

In this section, we exemplify how the L_1-SCCA procedure works in order to reduce the effect of nuisance variables, such as subject properties (e.g., age, sex), site properties, and scanning protocols (e.g., eyes open/close). This procedure allowed us to utilize data with a great variety of demographic distributions and imaging conditions from multiple imaging sites, for the construction of a classifier with good generalization capability across "foreign" sites. We begin by considering a simple and extreme artificial example to illustrate how L_1-SCCA can fulfill this role. Suppose that site X recruited almost exclusively ASD participants and only one TD participant and utilizes a closed-eye paradigm, and site Y recruited almost exclusively TD participants, only one ASD participant and utilizes an open-eye paradigm. In this case, it should be quite easy for any machine-learning

algorithm to classify ASD and TD based on the FCs associated with the eyes open/close condition, rather than the ASD/TD label. This is of course an undesirable situation and leads to very poor generalization across new imaging sites. However, when we use L_1-SCCA, at least one canonical variable is assigned to the eyes open/close condition (i.e. nuisance-related canonical variable), and at least another canonical variable is assigned to the ASD/TD label. By introducing the L_1-regularization canonical variables compete for the FCs. This reduces the number of FCs common across canonical variables. More specifically, the FCs assigned to the nuisance-related canonical variables are penalized, and the classifier uses only FCs directly associated with the ASD/TD-related canonical variables. Thus, artifactual effects by canonical variables other than the ASD/TD label are reduced in classification. The same argument applies to any other unevenly distributed attribute, including psychotic drugs and sex. In practice, an FC can be related to different demographic attributes simultaneously (Supplementary Fig. 7). However, as depicted in Supplementary Fig. 8C, the canonical variables assigned to the nuisance variables always have the strongest association with the nuisance-related FCs. Considering all these factors, we can safely assume that the L_1-SCCA procedure can effectively suppressed cross talk from nuisance variables.

Supplementary Note 7

Details about data standardization.

For L_1-SCCA, the standardization was conducted using only 8 out of 9 folds, and the testing pool for LOOCV was never used. Moreover, evaluating the classification performance of SLR, standardization is performed with a leave-one-subject-out (LOSO) approach. Concretely, the data standardization of the training set was done independently from the one of the test data. The test data is then standardized using the mean and standard deviation (SD) derived from the independent dataset. In the LOSO standardization, all-but-one USA subjects were concatenated to the Japanese dataset in order to find mean and SD. These parameters were subsequently used to standardize both the Japanese dataset (i.e. training set) and the remaining USA subject (i.e. test set, never used for standardization). It should be noted that even though a part of the USA dataset was used for standardizing the Japanese dataset, the actual learning was done using only the Japanese samples. The LOSO approach is useful because it removes the bias caused by the different scanning conditions between Japanese and USA dataset, leading to a better balance between Specificity and Sensitivity. Given that for each USA sample a slightly different mean and SD were used for standardization, the actual number of selected FCs is 15.96 ± 0.23 (99.6% overlap). In order to report information other than classification performance (e.g. the weights of the classifier, number of FCs), the whole USA dataset was concatenated to the Japanese dataset for standardization and the classifier was retrained, using only the Japanese dataset. This procedure led to the finally reported 16 FCs.

Supplementary References

1. Noirhomme, Q. *et al.* Biased binomial assessment of cross-validated estimation of classification accuracies illustrated in diagnosis predictions. *Neuroimage Clin* **4,** 687–694 (2014).
2. Zou, H. & Hastie, T. Regularization and Variable Selection via the Elastic Net. *Journal of the Royal Statistical Society: Series B (Statistical Methodology)* **67,** 301–320 (2005).
3. Whelan, R. *et al.* Neuropsychosocial profiles of current and future adolescent alcohol misusers. *Nature* **512,** 185–189 (2015).
